# Conformational Panorama of Cycloundecanone: A Rotational Spectroscopy Study

**DOI:** 10.1021/acs.jpca.2c04855

**Published:** 2022-08-23

**Authors:** Valerie W. Y. Tsoi, Ecaterina Burevschi, Shefali Saxena, M. Eugenia Sanz

**Affiliations:** Department of Chemistry, King’s College London, London SE1 1DB, United Kingdom

## Abstract

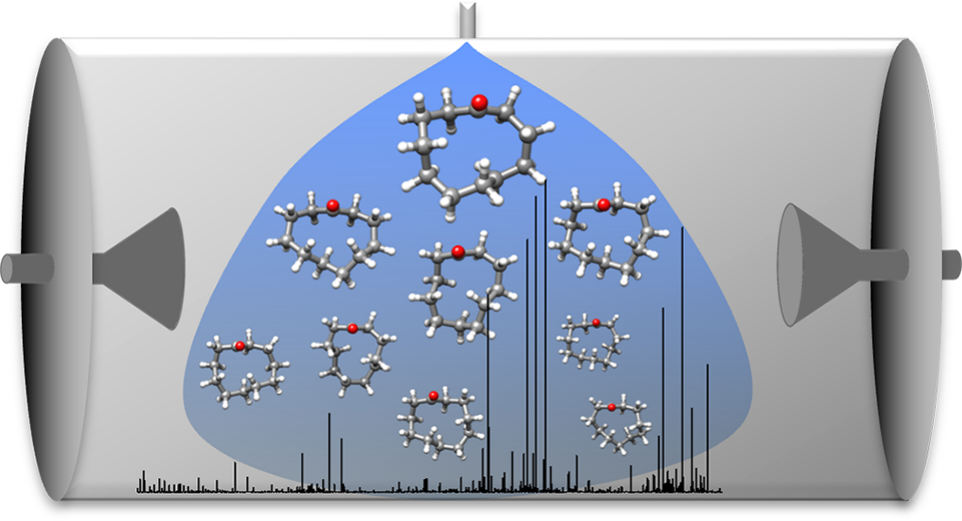

The conformational landscape of the medium-size cyclic ketone cycloundecanone has been investigated using chirped-pulse Fourier transform microwave spectroscopy and computational calculations. Nine conformations were observed in the rotational spectrum and identified from the comparison of experimental and theoretical rotational constants as well as the observed and predicted types of rotational transitions. All singly substituted ^13^C isotopologues were observed for the most abundant conformer, which allowed the determination of partial substitution and effective structures. The most abundant conformer dominates the rotational spectrum and is almost 40 times more abundant than the least abundant conformer. Conformational preferences are governed by the combination of transannular H···H and eclipsed HCCH interactions.

## 1 Introduction

Medium-size rings are defined as those containing 8–11 members.^1^ They are of interest due to their use as reagents in the synthesis of larger cycles,^2−5^ for their use in medicinal chemistry,^6^ and because they constitute a structural transition between small and common rings (3–7-membered rings) and large cycles (with 12 or more atoms in the ring). However, they have distinctive properties from the other type of cycles. Three- and four-membered rings show high torsional and angle strain. Five- to seven-membered rings have some torsional and angle strain, while large cycles have low levels of strain and the configuration of their chains resemble those of open-chain compounds.^1^ Medium-size rings are characterized by exhibiting low torsional and angular strain, like large cycles, but differently to other rings, they show steric strain. This takes the form of transannular interactions between the substituents of the atoms forming the ring, which are usually hydrogen atoms since larger substituents are pushed to point outside the ring. As a result, medium-size rings are usually quite reactive and difficult to synthesize, and they are expected to present more than one conformation.

Conformational analysis of medium-size rings has been undertaken using computational and experimental methods. Several conformations have been observed for cycloalkanes C_*n*_H_2*n*_, *n* = 8–11, using NMR spectroscopy, electron and X-ray diffraction in combination with molecular mechanics and quantum chemistry calculations.^7−14^ Similar reports have been published regarding cycloketones.^15−18^ The latter have the advantage of being polar and therefore amenable to study using rotational spectroscopy, which can provide a more detailed analysis than NMR spectroscopy or diffraction methods due to its high resolution and the direct relation of rotational spectra to structural parameters.^19^ Furthermore, rotational spectroscopy examines molecules in the gas phase, where they are free from crystal packing forces or interactions with solvents, and thus inherent conformational preferences are obtained.

To gain a better understanding of the conformational preferences of medium and large cycles, we have recently studied cyclooctanone^20^ and cyclododecanone^21^ by chirped-pulse Fourier transform microwave (CP-FTMW) spectroscopy. Three conformers were observed for cyclooctanone^20^ and seven for cyclododecanone.^21^ Both cycloketones showed a very strong preference for the lowest-energy conformation, which was driven by the minimization of transannular interactions, and, to a smaller degree, HCCH eclipsed configurations. Here, we extend our conformational studies of cycloketones to 11-membered cycloundecanone (CU).

CU is used in the synthesis of macrocycles such as cyclopentadecanone.^5^ It has been studied in condensed phases by X-ray crystallography and NMR spectroscopy.^22,23^ From the analysis of ^1^H and ^13^C NMR spectra, it was concluded that it appears as a single conformation, but its structure could not be obtained.^23^ Groth determined CU’s X-ray structure and established that it crystallizes in a monoclinic crystal system.^22^ Later on, a molecular mechanics study predicted the X-ray structure to be the lowest-energy one and reported its MM3 and MM4 structural parameters.^15^ No molecular mechanics structural data were reported for any other conformations.

CU is expected to present several conformers in the gas phase, as observed for cyclooctanone^20^ and cyclododecanone.^21^ Our investigation, using CP-FTMW spectroscopy and computational calculations, sought to characterize its conformational landscape and address the following questions: Would the conformational behavior of CU be closer to that of cyclooctanone (another medium-size ring), or cyclododecanone (of similar size)? Would its conformational preferences be driven by the same intramolecular interactions? Our results, as well as experimental and computational details, are described in the sections below.

## 2 Methods

### 2.1 Theoretical

The potential energy surface of CU was initially explored using the conformer-rotamer sampling program CREST,^24^ which yielded a first set of 83 conformations. Their structures were optimized at B3LYP-^25,26^ D3BJ^27,28^ and MP2^29^ levels, using Pople’s basis set 6-311++G(d,p),^30,31^ which resulted in 19 conformations within 12 kJ mol^–1^ (see Tables 1, S1 and S2). To minimize the chances of missing possible conformations due to imperfect sampling, a second mapping of the conformational landscape using CREST was performed taking the optimized structure of conformer **VI** (see Table 1) as starting structure. Conformer **VI** was chosen as it had a relative zero-point energy that was approximately halfway between the lowest-energy conformer and the highest-energy conformer within 1000 cm^–1^. This second search yielded the same set of conformers within 12 kJ mol^–1^ as the first search. All predicted conformations were confirmed to be local minima by running harmonic frequency calculations at the corresponding levels of theory and checking that there were no predicted imaginary frequencies.

**Table 1:** Experimental and Theoretical Spectroscopic Constants of the Observed Conformers of Cycloundecanone

	I			II			III		
	Exp.	B3LYP^j^	MP2^j^	Exp.	B3LYP	MP2	Exp.	B3LYP	MP2
*A*^a^ (MHz)	1074.99150(32)^i^	1074.0	1088.8	1048.49719(47)	1052.9	1062.1	1098.98025(34)	1101.4	1115.8
*B* (MHz)	873.62177(33)	870.2	875.0	872.78614(49)	868.0	873.4	815.94313(42)	810.1	813.9
*C* (MHz)	571.36275(30)	569.9	576.9	582.97207(71)	581.7	588.4	564.86445(43)	562.7	569.3
Δ_*J*_ (kHz)	0.0579(90)	–	–	0.084(15)	–	–	0.0395(57)	–	–
*Δ*_*JK*_ (kHz)	–0.051(10)	–	–	–0.101(29)	–	–	–	–	–
*δ*_*J*_ (kHz)	–	–	–	–	–	–	0.0162(42)	–	–
κ^b^	0.20	0.19	0.29	0.25	0.22	0.20	–0.06	–0.08	–0.10
*μ*_*a*_^c^ (D)	Y	0.5	0.4	Y	–0.8	–0.8	Y	–0.8	–0.7
*μ*_*b*_ (D)	Y	0.5	–0.4	N	0.2	–0.1	N	–0.2	0.2
*μ*_*c*_ (D)	Y	2.4	2.2	Y	2.6	2.4	Y	2.6	2.4
σ^d^ (kHz)	5.7	–	–	5.7	–	–	5.0	–	–
*N*^e^	65	–	–	37	–	–	44	–	–
Δ*E*^f^ (cm^–1^)	–	0.0	0.0	–	353.3	500.7	–	365.8	497.1
Δ*E* + ZPC^g^(cm^–1^)	–	0.0	0.0	–	250.4	431.1	–	355.3	450.8
Δ*G*^h^ (cm^–1^)	–	0.0	0.0	–	56.2	305.9	–	192.5	271.3

	IV			V			VI		
	Exp.	B3LYP	MP2	Exp.	B3LYP	MP2	Exp.	B3LYP	MP2
*A*^a^ (MHz)	1150.01667(46)^i^	1153.9	1163.2	1043.96411(49)	1047.5	1056.7	1052.06849(59)	1056.0	1063.1
*B* (MHz)	829.44239(34)	824.1	831.2	865.15355(57)	859.9	866.4	873.30667(62)	868.2	874.6
*C* (MHz)	563.39249(22)	561.9	567.8	560.68924(74)	559.4	565.8	564.45246(78)	563.6	568.8
*Δ*_*J*_ (kHz)	–	–	–	0.028(11)	–	–	0.111(23)	–	–
*Δ*_*JK*_ (kHz)	–	–	–	–	–	–	–0.146(43)	–	–
κ^b^	–0.09	–0.11	–0.12	0.26	0.23	0.22	0.27	0.24	0.24
*μ*_*a*_^c^ (D)	N	–0.1	–0.1	N	–0.1	0.0	Y	0.7	0.7
*μ*_*b*_ (D)	Y	–0.4	0.4	Y	1.4	–1.3	Y	0.8	–0.8
*μ*_*c*_ (D)	Y	2.3	2.1	Y	–2.4	2.3	Y	2.4	2.2
σ^d^ (kHz)	7.4	–	–	6.1	–	–	4.8	–	–
*N*^e^	26	–	–	27	–	–	28	–	–
Δ*E*^f^ (cm^–1^)	–	672.0	777.8	–	643.6	862.2	–	619.2	832.1
Δ*E* + ZPC^g^ (cm^–1^)	–	532.9	643.9	–	562.3	754.6	–	580.3	790.3
Δ*G*^h^ (cm^–1^)	–	291.5	463.5	–	407.6	566.5	–	404.3	628.4

	VII			VIII			IX		
	Exp.	B3LYP	MP2	Exp.	B3LYP	MP2	Exp.	B3LYP	MP2
*A*^a^ (MHz)	1044.38009(62)^i^	1045.2	1059.8	1131.11965(43)	1123.7	1138.0	1088.20190(53)	1088.1	1102.2
*B* (MHz)	867.56948(76)	865.6	867.0	779.83784(52)	779.1	782.6	844.96372(49)	841.3	843.1
*C* (MHz)	573.3155(50)	573.3	578.8	543.56556(84)	538.5	544.0	562.50620(50)	560.4	565.6
*Δ*_*J*_ (kHz)	0.087(18)	–	–	0.052(14)	–	–	–	–	–
κ^b^	0.25	0.24	0.20	–0.20	–0.18	–0.20	0.07	0.06	0.03
*μ*_*a*_^c^ (D)	N	–0.5	–0.4	Y	–1.7	–1.5	N	–0.3	0.3
*μ*_*b*_(D)	N	0.3	–0.3	N	0.5	0.5	Y	–1.2	–1.1
*μ*_*c*_(D)	Y	2.6	2.4	Y	–2.2	2.1	Y	2.4	2.2
σ^d^ (kHz)	3.3	–	–	5.6	–	–	5.7	–	–
*N*^e^	15	–	–	20	–	–	19	–	–
Δ*E*^f^ (cm^–1^)	–	675.8	820.5	–	635.5	816.2	–	765.8	1060.4
Δ*E* + ZPC^g^(cm^–1^)	–	629.0	764.0	–	652.1	784.4	–	710.4	967.7
Δ*G*^h^ (cm^–1^)	–	437.2	580.5	–	463.7	561.9	–	564.0	773.0

a *A, B*, and *C* are the rotational constants; *Δ*_*J*_*, Δ*_*JK*_, and *δ*_*J*_ are quartic centrifugal distortion constants.

b Ray’s asymmetry parameter.

c *μ*_*a*_*, μ*_*b*_, and *μ*_*c*_ are the electric dipole moments along the principal inertial axes *a*, *b* and *c*, respectively. For the experimental column, it is indicated whether the corresponding transition was observed (Y) or no (N).

d Rms deviation of the fit.

e Number of fitted transitions.

f Relative electronic energies.

g Relative electronic energies including the zero-point correction.

h Free Gibbs energies at 367.15 K.

i Standard error in parentheses in units of the last digit.

j B3LYP-D3BJ/6-311++G(d,p) and MP2/6-311++G(d,p) levels of theory.

### 2.2 Experimental Section

**Figure 1:**
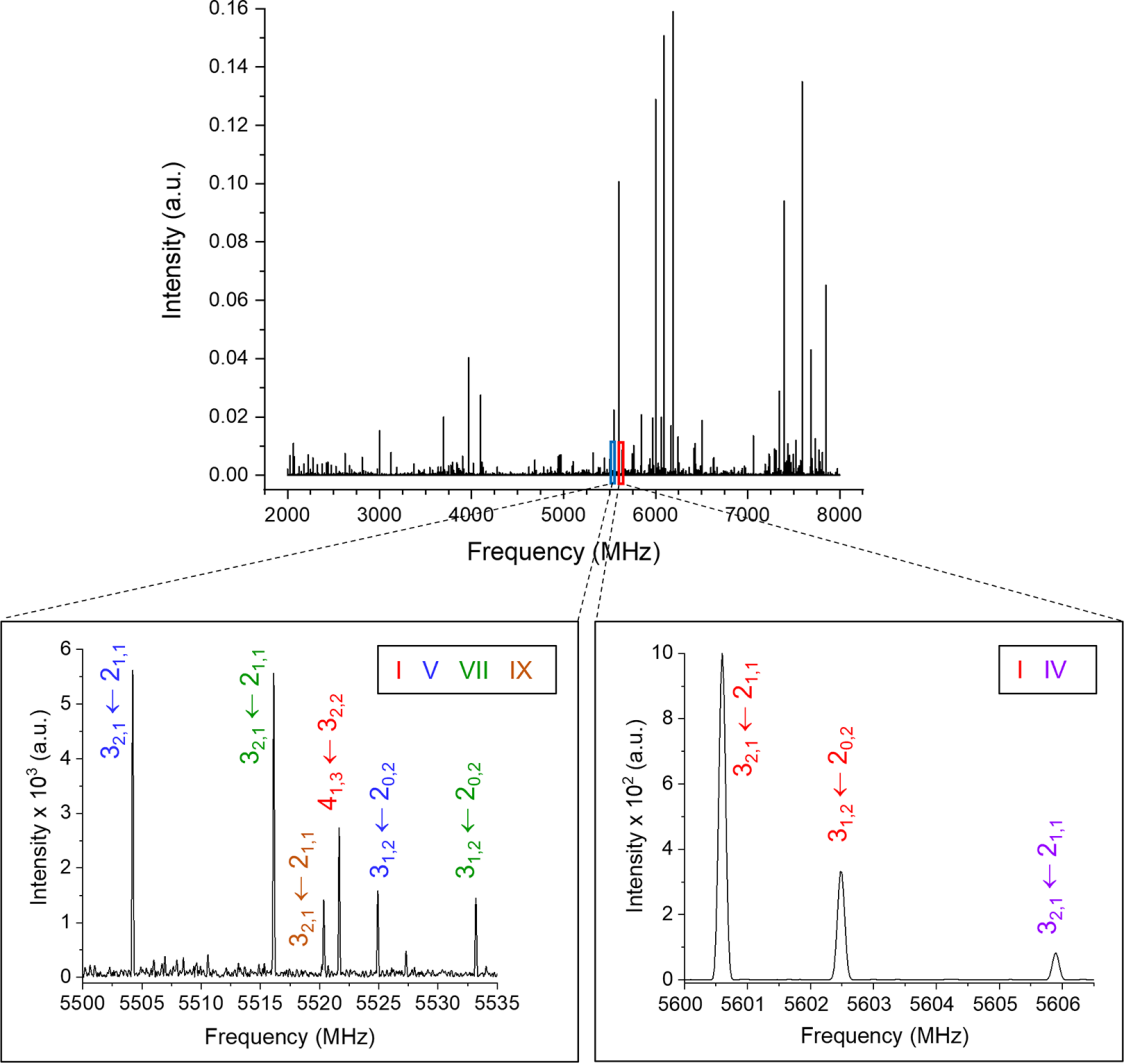
Broadband rotational spectrum of cycloundecanone. The enlarged sections show representative transitions of some of the conformers.

The rotational spectrum of CU (Figure 1) was recorded using a chirped-pulse Fourier transform microwave spectrometer that operates in the 2–8 GHz frequency range.^32,33^ A sample of CU was purchased (≥96%, Santa Cruz Biotechnology) and used without further purification. CU was heated to 367 K, seeded in Ne at a backing pressure of 6 bar, and introduced into the vacuum chamber of our instrument through a pinhole nozzle of 1 mm diameter as a supersonic jet. Molecular pulses of 1000–1100 μs were found to be optimal. The molecules of CU in the jet were polarized using 4 μs long chirped microwave pulses, spaced 30 μs between them. Four microwave pulses were applied per molecular pulse. After each microwave pulse, the molecular free induction decay was collected for 20 μs, stored in a fast oscilloscope in the time domain, and Fourier transformed to the frequency domain for analysis. The final rotational spectrum has 5.2 MFIDs.

## 3 Results and Discussion

### 3.1 Rotational Spectrum

All the lower-energy conformers of CU are predicted to have *μ*_*c*_ larger than 2 D. Therefore we started looking for *c*-type transitions of the series *J* + 1 ← *J* with *K*_–1_ = 0, 1 for the two lowest-energy conformers. We found one intense and one weaker set of lines close to their predicted frequencies and with the expected patterns. After preliminary rotational constants were obtained from initial fits, measurement of additional transitions confirmed the assignments and resulted in the experimental rotational constants of columns 1 and 4 of Table 1. The fits were performed using Pickett’s programs,^34^ and the Watson semirigid Hamiltonian in the A reduction and the III^l^ representation^35^ since both species are oblate tops. From the comparison between experimental and theoretical rotational constants, the observed species correspond to conformers **I** and **II**. The measured transitions (*a*-, *b*-, and *c*-type transitions for conformer **I** and *a*- and *c*-type transitions for conformer **II**), are consistent with the values of the dipole moment components predicted for each conformer (see Table 1).

Once the first two conformers were assigned, we removed their lines from the spectrum and searched for additional conformers using the automated fitting tool in PGOPHER,^36,37^ which is based on the AUTOFIT algorithm.^38^ After several cycles, where one new species will be found, their transitions measured and then removed from the spectrum, and another species searched for in a new cycle, we identified seven other conformers of CU (Table 1).

Conformer identification was straightforward as there is very close agreement between the theoretical rotational constants predicted by both B3LYP-D3BJ and MP2 methods and the experimental rotational constants (Table 1). Identification was corroborated for each conformer by the type of transitions observed (*a*-, *b*-, or *c*-type transitions), their relative intensities, and their consistency with predictions of dipole moment components *μ*_*a*_, *μ*_*b*_, and *μ*_*c*_. In our experiment, line intensity is proportional to the number density of a species and to the square of the corresponding dipole moment component, that is, *I*_*i*_ = *N*_*i*_*μ*_*ij*_^2^, where *I*_*i*_ is the observed line intensity of conformer *i*, *N*_*i*_ is its number density, and *μ*_*ij*_ is the dipole moment component of conformer *i* along the *j* = *a*, *b*, or *c* principal inertial axis. Thus, low values of *μ*_*ij*_ are likely to result in the non-observation of the corresponding *j*-type transitions. For example, for conformer **III**, where *μ*_*b*_ is predicted to be 0.2 D, no *b*-type transitions have been observed.

Conformational relative abundances can be estimated from the expression above relating number density and line intensity, assuming that all conformers are in their ground vibrational states.^39^ Since all conformers have *c*-type spectrum, and their *μ*_*c*_ are predicted to be very similar, we calculated their relative abundances considering the theoretical B3LYP-D3BJ dipole moments and the intensity of their common *c*-type transitions. The values we obtained are **I**:**II**:**III**:**IV**:**V**:**VII**:**VIII**:**VI**:**IX** = 39.6:6.3:5.0:4.4:1.9:1.9:1.2:1.0:1.0. Conformer **I** is approximately 6 times more abundant than the next conformer in abundance, conformer **II**, and about 8 times more abundant than conformer **III**, the third most abundant conformer.

### 3.2 Substitution and Effective Structures of the Most Abundant Conformer

**Figure 2:**
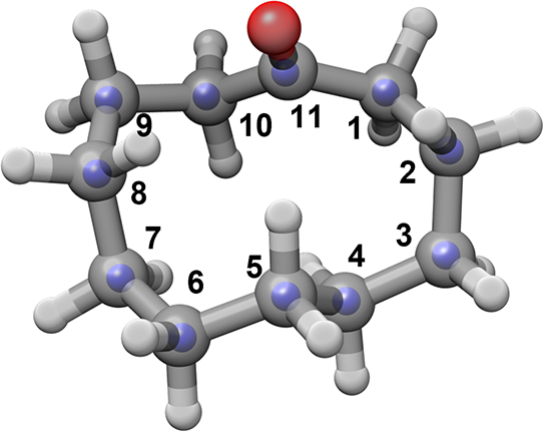
Overlay of the B3LYP-D3BJ/6-311++G(d,p) structure of conformer **I** of cycloundecanone (gray framework) and the substitution coordinates of the carbon atoms (blue spheres).

The rotational spectrum of conformer **I** was very intense (its average signal-to-noise ratio was about 340), which made it possible to observe all the singly substituted ^13^C species in their natural abundance (1.1%) and determine their rotational constants (Table S3). The ^18^O isotopologue was too weak to be observed due to its lower natural abundance of 0.2%, which would correspond to a S/N ratio of approximately 0.7. The differences between the ^13^C isotopologues’ experimental moments of inertia and those of the parent species were used to determine the coordinates of the C atoms in their principal inertial axis system by applying Kraitchman’s equations^40^ within the program KRA^41^ (see Table S4). The *r*_s_ substitution structure was then determined from the carbon atoms’ coordinates (Table 2 and Figure 2).

**Table 2:** Substitution, Effective and Equilibrium Bond Lengths (Å), Angles (deg), and Dihedral Angles (deg) of Conformer **I** of Cycloundecanone

Parameter	*r*_s_^a^	*r*_0_^b^	*r*_e_^d^	*r*_e_^e^	X-ray^f^
*r*(C_1_–C_2_)	1.585(21)	1.5421(95)	1.545	1.543	1.540(3)
*r*(C_2_–C_3_)	1.474(25)	1.5417(93)	1.538	1.536	1.524(3)
*r*(C_3_–C_4_)	1.5322(49)	1.5284(50)	1.534	1.532	1.528(3)
*r*(C_4_–C_5_)	1.536(16)	1.5357(80)	1.532	1.530	1.525(3)
*r*(C_5_–C_6_)	1.509(23)	1.5304(65)	1.538	1.537	1.539(3)
*r*(C_6_–C_7_)	1.535(11)	1.5416(52)	1.538	1.537	1.542(3)
*r*(C_7_–C_8_)	1.500(54)	1.5450(50)^c^	1.545	1.543	1.539(3)
*r*(C_8_–C_9_)	1.575(57)	1.5403(56)	1.537	1.536	1.530(3)
*r*(C_9_–C_10_)	1.5317(59)	1.5269(61)	1.533	1.532	1.530(3)
*r*(C_10_-C_11_)	1.462(39)	1.5215(50)	1.519	1.520	1.516(3)
*r*(C_11_–C_1_)	1.511(19)	1.5274(43)^c^	1.526	1.526	1.518(3)
∠(C_1_–C_2_-C_3_)	114.96(72)	114.06(74)	114.5	113.7	114.6(2)
∠(C_2_–C_3_-C_4_)	116.03(22)	115.69(18)	116.0	115.1	116.0(2)
∠(C_3_–C_4_-C_5_)	115.91(85)	115.41(50)	115.0	114.5	115.6(2)
∠(C_4_–C_5_-C_6_)	114.09(81)	113.80(31)^c^	113.8	113.2	114.0(2)
∠(C_5_–C_6_-C_7_)	114.31(33)	114.38(12)	114.7	114.1	114.2(2)
∠(C_6_–C_7_-C_8_)	113.1(15)	113.27(28)	113.8	113.0	113.8(2)
∠(C_7_–C_8_-C_9_)	115.50(85)	115.44(29)	115.4	114.9	115.4(2)
∠(C_8_–C_9_-C_10_)	113.64(62)	114.05(13)	114.4	114.0	113.8(2)
∠(C_9_–C_10_-C_11_)	116.3(18)	114.55(28)	114.9	114.1	114.6(2)
∠(C_10_-C_11_–C_1_)	123.7(39)	118.96(43)^c^	118.2	118.5	119.5(2)
∠(C_11_–C_1_-C_2_)	114.8(15)	112.99(44)^c^	113.1	112.8	113.9(2)
τ(C_1_–C_2_-C_3_–C_4_)	–55.03(90)	–54.5(11)	–54.1	–53.8	–55.7(3)
τ(C_2_–C_3_-C_4_–C_5_)	–62.28(86)	–63.28(96)	–63.0	–64.2	–64.6(3)
τ(C_3_–C_4_-C_5_–C_6_)	175.93(29)	175.65(22)	175.6	177.4	173.7(2)
τ(C_4_–C_5_-C_6_–C_7_)	–65.9(1.0)	–64.80(31)^c^	–64.8	–64.3	–63.6(3)
τ(C_5_–C_6_-C_7_–C_8_)	–64.9(15)	–65.00(33)^c^	–65.0	–65.1	–64. 0(3)
τ(C_6_–C_7_-C_8_–C_9_)	137.52(65)	137.51(18)	136.2	137.6	139.8(2)
τ(C_7_–C_8_-C_9_–C_10_)	–58.69(76)	–59.17(18)	–59.5	–59.6	–61.4(3)
τ(C_8_–C_9_-C_10_-C_11_)	–59.4(13)	–58.90(33)^c^	–58.9	–58.0	–59.6(3)
τ(C_9_–C_10_-C_11_–C_1_)	162.01(62)	162.69(21)^c^	163.0	163.7	159.6(2)
τ(C_10_-C_11_–C_1_-C_2_)	–127.1(14)	–130.22(48)^c^	–129.1	–130.8	–127.2(2)
τ(C_11_–C_1_-C_2_–C_3_)	90.7(18)	93.43(61)^c^	92.8	92.7	95.1(3)

a The substitution structure has been determined from the atomic coordinates including Costain’s error, and with signs taken from the B3LYP-D3BJ calculated structure.

b Effective structure; nonfitted parameters were fixed to the B3LYP-D3BJ/6-311++G(d,p) values.

c Derived from the determined *r*_0_ structure, not fitted directly.

d B3LYP-D3BJ/6-311++G(d,p) parameters.

e MP2/6-311++G(d,p) parameters.

f Data taken from Table XX in the Supporting Information of ref (15).

An alternative method of calculating molecular structures involves performing a least-squares fit of the experimental moments of inertia of the parent and isotopic species, to determine the effective *r*_0_ structure. This is compared with the *r*_s_, the *r*_e_ (both MP2 and B3LYP-D3BJ), and the X-ray structures in Table 2.

The *r*_0_ structure shows an excellent agreement with both *r*_e_ structures, both for bond lengths and angles. The C–C bond lengths involving the carbonyl carbon are slightly shortened with respect to typical C–C values, and this behavior is consistent for *r*_0_ and *r*_e_ structures. In contrast, the *r*_s_ structure presents some C–C distances that are somewhat too long or too short, with values quite different from *r*_0_ and *r*_e_ structures, which may be due to known shortcomings in calculating substitution structures. In the case of bond lengths involving C_11_, this could be attributed to its imaginary coordinate *c*. Bond lengths involving C_2_, C_5_, and C_8_ are affected by large uncertainties in some of the principal axis coordinates of these atoms (see Table S4). Substitution angles and dihedrals mostly agree with those of *r*_0_ and *r*_e_ structures, except for those involving C_11_, which show differences of up to 4.7° (∠(C_10_–C_11_–C_1_)) and 3.1° (τ(C_10_–C_11_–C_1_–C_2_)). There is a good agreement of the *r*_0_ with the X-ray structure, with differences mostly in the dihedral angles. This indicates that there are no strong perturbations induced by the lattice in the condensed phase structure.

Values of the *r*_0_ bond lengths in conformer **I** of CU are very similar to the *r*_0_ ones of conformer **I** of cyclododecanone. The ∠CCC bond angles of CU vary between 113 and 116°. Although they are similar to cyclododecanone, on average they are slightly more obtuse. Removing the highest and lowest *r*_0_ values, the average angle for cyclododecanone is 114.1°, while for CU is 114.5°. The dihedral angles of CU deviate more from +60°, −60°, and 180° than those of the global minimum of cyclododecanone, indicating a higher torsional strain.

### 3.3 Discussion

The observed conformers are predicted to be among the lowest in energy by both B3LYP-D3BJ and MP2 methods. From our experimental results, the conformers can be divided into three groups: conformer **I**, considerably more abundant than the rest; conformers **II**–**IV**, with abundances between 11 and 16% of conformer **I**; and conformers **V–IX** with abundances between 2 and 5% of conformer **I**. These observations are consistent with theoretical predictions. Both methods predict conformer **I** to be the global minimum and agree that conformers **II**–**IV** are next in energy, followed by **V–IX**, although there are some disagreements on the energy ordering of the last five conformers. MP2 predicts a larger energy gap between conformers **I** and **II** than B3LYP-D3BJ and also a larger difference in Gibbs energies, which is in agreement with the higher abundance observed for conformer **I**.

The experimental conformational abundances can be compared to the theoretical ones calculated at the pre-expansion temperature of 367.15 K using Gibbs free energies, which are **I**:**II**:**III**:**IV**:**V**:**VII**:**VIII**:**VI**:**IX** = 9.1 : 7.3 : 4.3 : 2.9 : 1.8 : 1.6 : 1.5 : 1.9 : 1.0 from B3LYP-D3BJ and **I**:**II**:**III**:**IV**:**V**:**VII**:**VIII**:**VI**:**IX** = 20.7 : 6.2 : 7.1 : 3.4 : 2.2 : 2.1 : 2.3 : 1.8 : 1.0 from MP2. These values roughly reproduce the experimental trend, with MP2 results in better agreement with experimental abundances. Differences between theoretical and experimental conformational distributions can be the result of relaxation from higher-energy conformers to lower-energy ones. This process takes place by collisions with the carrier gas in the supersonic jet, if interconversion barriers between conformers are sufficiently low.^42,43^

The theoretical rotational constants are in very close agreement with the experimental ones. The average deviations are 0.4% for B3LYP-D3BJ and 0.7% for MP2. We have thus taken the B3LYP-D3BJ structures to discuss the intramolecular interactions in the observed conformers of CU and rationalize the observed conformational preferences. As a medium-size cycle, CU is expected to have low angle and torsional strain but higher levels of steric strain due to transannular interactions. Looking at the B3LYP-D3BJ bond angles of all observed conformations (Table S5), they are mostly in the range 112°–118°, with averages around 115°. Conformers **VIII** and **IX**, among the least abundant ones, show one angle with larger values of 119.2° and 120.3°, respectively. Bond angles are similar to those of cyclooctanone^20^ and cyclododecanone^21^ and not expected to contribute significantly to energy differences between conformations.^1^ The dihedral angles of the most abundant conformer are, in their majority, very close to the ideal 60°, −60° (gauche), and 180° (anti). However, the dihedrals of the rest of the conformers are further from 60° and 180°, although there is not a clear trend. For example, the conformer with larger dihedral deviations is conformer **IV**, which is the fourth in abundance. Conformer **VI**, one of the least abundant, has ∠CCCC angles with similar differences from the ideal values to conformer **I**. In general, the dihedral angles of CU conformers show larger differences from 60°, −60° and 180° than cyclododecanone’s, which could be a consequence of trying to relieve steric strain due to transannular interactions and eclipsed configurations (Table S5).

**Figure 3:**
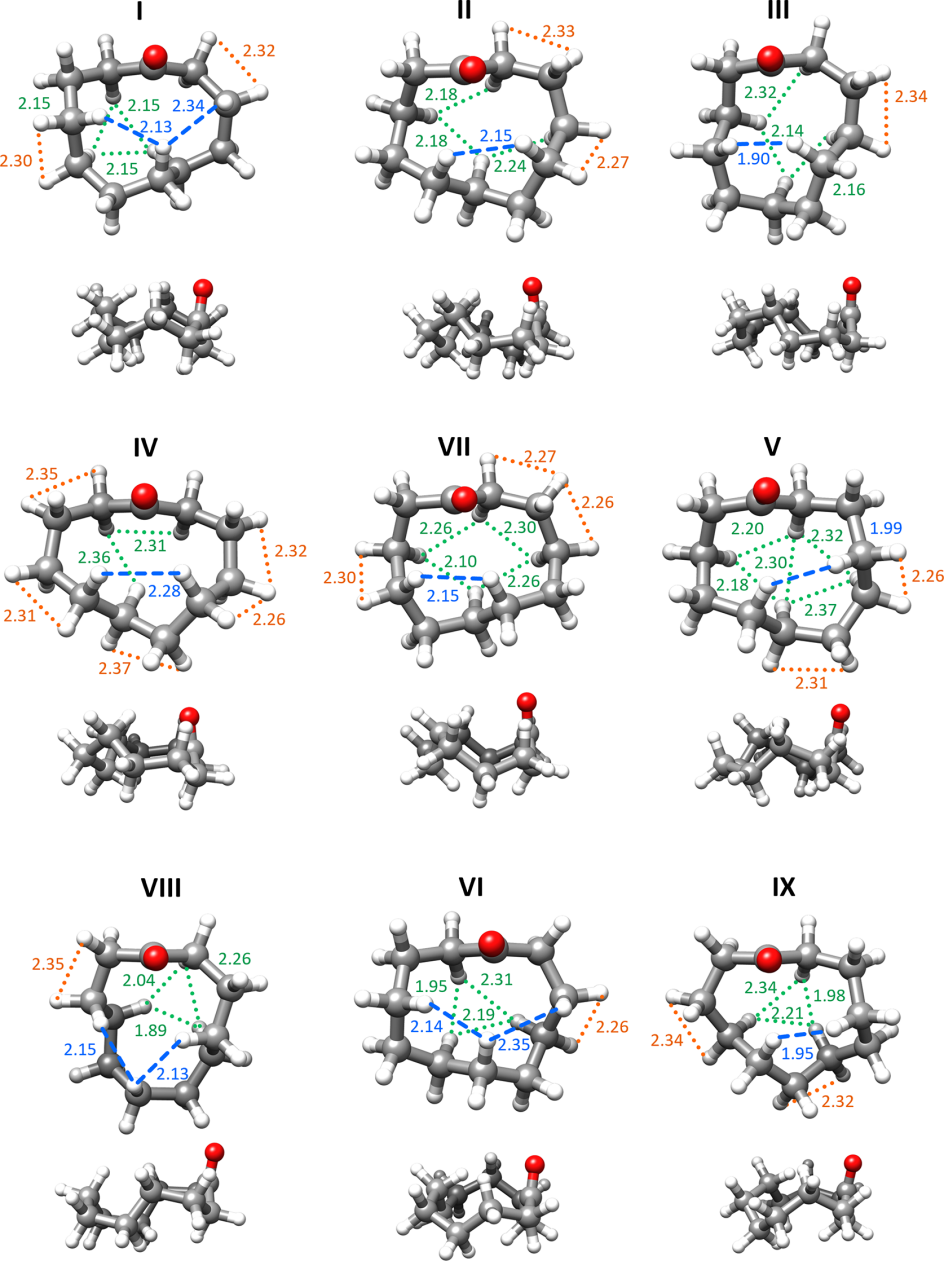
Observed conformers of cycloundecanone (top and side views) in decreasing order of abundance, indicating the transannular H···H interactions above the plane of the ring (blue), below (green), and the eclipsed interactions (orange).

In all conformers of CU there are interactions between H atoms across the ring (transannular) and between hydrogens in adjacent C atoms (eclipsed, HCCH) where the atoms are closer than the sum of their van der Waals radii, 2.40 Å.^44^ We have considered eclipsed interactions where the dihedral HCCH angles are below 40°, and those, together with transannular interactions, are shown in Figure 3. The observed conformers show one or two transannular interactions above the ring, while interactions below the ring range from two to five. A fine balance between these interactions seems to determine conformational abundances and relative energies. Conformer **I**, the most abundant, presents five transannular interactions, two above and three below the ring, and two HCCH interactions. The number of contacts as well as their values are similar to those of conformer **II**, but torsional strain is lower in **I**, which has ∠CCCC dihedral angles closer to 60°, −60° and 180°. Moreover, one of the ∠HCCH angles in **II** has a small value of 12.9°. Conformer **III** has fewer transannular H···H interactions than **I** or **II**, but one of them is very short at 1.90 Å, and it also shows higher deviations of ∠CCCC dihedrals from ideal values. Conformer **IV** only has three transannular interactions, but five HCCH eclipsed interactions (one with ∠HCCH = 8.4°). These factors explain the lower abundance and higher predicted energies of conformers **II**, **III**, and **IV** relative to **I**. Conformers **V** and **VII** have the same number of H···H interactions as **IV**, but they are shorter, accounting for their lower abundance. The least abundant conformers (**VI**, **VIII**, and **IX**) all have one transannular interaction with a distance of 1.95 Å or lower. Conformer **VI** has one eclipsed interaction with a very acute angle of ∠HCCH = 3.8°.

We performed a noncovalent interaction (NCI) analysis^45,46^ to visualize the interactions in cycloundencanone’s conformers. This examines the derivatives of the electron density and its reduced gradient and yields the regions where their values are low, which are characteristic of NCI, as reduced gradient isosurfaces. In CU, the isosurfaces related to transannular H···H interactions are all a mixture of weak attractive and weak repulsive interactions, with greenish/yellowish colors, from which is difficult to draw conclusions (Figure S2). The NCI analysis also revealed attractive interactions between the carbonyl oxygen and some of the hydrogen atoms nearby. These involve the hydrogens on the C atom that is two C–C bonds apart from the carbonyl group, and in some cases hydrogens across the ring (see also Figure S3). These interactions do not seem to determine the cycle shape or to follow a pattern and thus do not appear to have a relevant role on the stability of the conformers, even though they contribute to it. In this, CU behaves like cyclooctanone and cyclododecanone.

CU presents a higher number of transannular and eclipsed interactions than cyclooctanone as well as shorter H···H distances. However, the number of transannular interactions is lower than in cyclododecanone, which exhibited 7 in most of the observed conformers. In comparison with cyclododecanone, CU has a higher number of conformers with transannular distances shorter than 2.0 Å and shows higher deviations of the ∠CCCC dihedral angles from ideal values. These are signs of higher steric strain.

The most common ring configuration for the observed conformers of CU has 3, 3, 3, and 2 C–C bonds per side. There is a lower variety of ring shapes than observed in cyclododecanone, probably due to the odd number of atoms and smaller ring size. Like in cyclododecanone, the carbonyl group in all conformers is approximately perpendicular to the plane of the ring.

Conformer **I** is the dominant structure for CU, being approximately 6 times more abundant than conformer **II**. In this, CU follows the same behavior observed for the even-membered ketones cyclooctanone^20^ and cyclododecanone,^21^ which also showed one main conformation in the gas phase. While in cyclooctanone^20^ and cyclododecanone^21^ the predominant conformers were roughly 40 times and 19 times, respectively, more abundant than the second ones in abundance, in CU the difference is notably reduced, as conformer **II** is only about 6 times less abundant than conformer **I**. This is consistent with the smaller energy gap predicted between them in CU (see Table 1).

## 4 Conclusions

Nine distinct conformations of CU have been identified from a rotational spectroscopy investigation in combination with quantum chemistry calculations. The lowest-energy predicted conformer was found to be predominant, with an abundance six times larger than the second most abundant conformer and almost 40 times larger than the least abundant conformers. The conformational preferences were found to be determined by minimization of transannular and eclipsed hydrogen atom interactions, similar to the behavior exhibited by cyclododecanone. However, eclipsed HCCH interactions seem to have a higher influence in CU, which is reflected in the higher differences of the ∠CCCC dihedrals from ideal values.

The structural and conformational data on CU presented here lay the groundwork for further studies of this ketone, for example, on their interactions with other molecules forming complexes or undergoing different reaction pathways.^47^ In this respect, cyclooctanone has been found to react with water, producing a geminal diol upon addition of one and two water molecules in the gas phase.^48^ Since CU is more reactive, it will be of interest to see if it undergoes a similar reaction forming a geminal diol upon hydration.

The behavior of CU resembles more closely that of cyclododecanone than of cyclooctanone, considering the number of low-energy conformations observed, the interactions they present, and the arrangement of the carbonyl group. The similarity in size between the 11- and 12-membered cycloketones appears to be more relevant to describe them than their classification as medium-size rings or macrocycles. Would the similarities between odd- and even-membered cycles increase as the size of the ring grows? Would there be features specifically associated with odd- or even-membered cycles? Further structural studies of larger cycles are necessary to establish their similarities and differences. Investigations of other cycles with different functional groups will help examine how conformational preferences and intramolecular interactions are affected. In this respect, rotational spectroscopic studies have already unveiled the behavior of crown ethers.^49−51^ Understanding the conformations and interactions of medium-size and large cycles is of interest to tune their properties and reaction mechanisms.

## Supporting Information Available

Theoretical spectroscopic constants and equilibrium structural parameters of all predicted conformers within 1000 cm^–1^, experimental spectroscopic constants of observed isotopologues, substitution coordinates, details of NCI analysis and C–H···O interactions, measured frequencies and residuals of observed rotational transitions (PDF)
