# Supplementary material for: Conformational Panorama of Cycloundecanone: A Rotational Spectroscopy Study
Source: J Phys Chem A. 2022 Aug 23;126(36):6185–93. doi: 10.1021/acs.jpca.2c04855 (PMC9483976; doi:10.1021/acs.jpca.2c04855)
Supplement: Supplementary file 1 — jp2c04855_si_001.pdf [file jp2c04855_si_001.pdf]

Conformational Panorama of Cycloundecanone: a Rotational Spectroscopy Study

Valerie W. Y. Tsoi, Ecaterina Burevschi, Shefali Saxena, M. Eugenia Sanz*

Department of Chemistry, King's College London, London SE1 1DB, UK

Table S1. B3LYP-D3BJ/6-311++G(d,p) spectroscopic constants of the conformers of cycloundecanone within 1000 cm^{-1}.

Parameter	I	II	III	IV	V	VI	VII	VIII
A^a (MHz)	1074.0	1052.9	1101.4	1153.9	1047.5	1056.0	1045.2	1123.7
B (MHz)	870.2	868.0	810.1	824.1	859.9	868.2	865.6	779.1
C (MHz)	569.9	581.7	562.7	561.9	559.4	563.6	573.3	538.5
κ	0.19	0.22	-0.08	-0.11	0.23	0.24	0.24	-0.18
μ_a (D)	0.5	-0.8	-0.8	-0.1	-0.1	0.7	-0.5	-1.7
μ_b (D)	0.5	0.2	-0.2	-0.4	1.4	0.8	0.3	0.5
μ_c (D)	2.4	2.6	2.6	2.3	-2.4	2.4	2.6	-2.2
ΔE^b (cm^{-1})	0.0	353.3	365.8	672.0	643.6	619.2	675.8	635.5
ΔE+ZPC^c (cm^{-1})	**0.0**	**250.4**	**355.3**	**532.9**	**562.3**	**580.3**	**629.0**	**652.1**
ΔG^d (cm^{-1})	**0.0**	**56.2**	**192.5**	**291.5**	**407.6**	**404.3**	**437.2**	**463.7**

Parameter	IX	X	XI	XII	XIII	XIV	XV
A^a (MHz)	1088.1	1148.0	1149.6	1221.9	1111.1	1048.0	1133.8
B (MHz)	841.3	772.9	777.8	785.0	842.3	863.8	774.5
C (MHz)	560.4	547.2	543.6	563.2	584.4	554.7	541.2
κ	0.06	-0.25	-0.23	-0.33	-0.02	0.25	-0.21
μ_a (D)	-0.3	-1.2	-1.4	-0.2	-0.4	-1.2	-0.7
μ_b (D)	-1.2	0.3	0.1	-0.7	-0.3	0.5	-0.3
μ_c (D)	2.4	-2.5	2.5	2.3	-2.5	-2.2	2.6
ΔE^b (cm^{-1})	765.8	734.6	768.4	830.2	767.0	829.2	904.0
ΔE+ZPC^c (cm^{-1})	**710.4**	**714.6**	**736.8**	**749.3**	**767.5**	**856.8**	**859.7**
ΔG^d (cm^{-1})	**564.0**	**556.1**	**549.8**	**645.0**	**654.3**	**767.7**	**698.6**

[a] *A, B* and *C* are the rotational constants; κ is Ray's asymmetric parameter; μ_a, μ_b, and μ_c are the electric dipole moments along the principal axes *a*, *b* and *c*, respectively. [b] Relative electronic energies. [c] Relative electronic energies including the zero-point correction. [d] Free Gibbs energies at 367.15 K.

Table S2. MP2/6-311++G(d,p) spectroscopic constants of the conformers of cycloundecanone within 1000 cm^{-1}.

Parameter	I	II	III	IV	X	XI	V	VII
A^a (MHz)	1088.8	1062.1	1115.8	1163.2	1156.0	1156.7	1056.7	1059.8
B (MHz)	875.0	873.4	813.9	831.2	783.1	792.0	866.4	867.0
C (MHz)	576.9	588.4	569.3	567.8	554.5	553.9	565.8	578.8
κ	0.29	0.20	-0.10	-0.12	-0.24	-0.21	0.22	0.20
μ_a (D)	0.4	-0.8	-0.7	-0.1	-1.0	-1.1	0.0	-0.4
μ_b (D)	-0.4	-0.1	0.2	0.4	0.4	0.0	-1.3	-0.3
μ_c (D)	2.2	2.4	2.4	2.1	2.4	2.3	2.3	2.4
ΔE^b (cm^{-1})	0.0	500.7	497.1	777.8	702.3	720.3	862.2	820.5
ΔE+ZPC^c (cm^{-1})	**0.0**	**431.1**	**450.8**	**643.9**	**675.8**	**707.8**	**754.6**	**764.0**
ΔG^d (cm^{-1})	**0.0**	**305.9**	**271.3**	**463.5**	**545.0**	**579.6**	**566.5**	**580.5**

Parameter	VIII	VI	XII	XV	XIV	IX	XIII
A^a (MHz)	1138.0	1063.1	1236.6	1056.7	1051.5	1102.2	1134.1
B (MHz)	782.6	874.6	789.2	866.4	878.7	843.1	841.1
C (MHz)	544.0	568.8	569.2	565.8	563.5	565.6	590.0
κ	-0.20	0.24	-0.34	0.22	0.29	0.03	-0.08
μ_a (D)	-1.5	0.7	-0.2	0.5	1.0	0.3	0.4
μ_b (D)	0.5	-0.8	0.7	-0.2	0.5	-1.1	0.2
μ_c (D)	2.1	2.2	2.1	2.5	-2.1	2.2	2.3
ΔE^b (cm^{-1})	816.2	832.1	884.1	904.2	837.3	1060.4	1060.1
ΔE+ZPC^c (cm^{-1})	**784.4**	**790.3**	**821.3**	**830.7**	**832.7**	**967.7**	**994.9**
ΔG^d (cm^{-1})	**561.9**	**628.4**	**750.2**	**638.5**	**715.0**	**773.0**	**800.4**

[a] *A, B* and *C* are the rotational constants; κ is Ray's asymmetric parameter; μ_a, μ_b, and μ_c are the electric dipole moments along the principal axes *a*, *b* and *c*, respectively. [b] Relative electronic energies. [c] Relative electronic energies including the zero-point correction. [d] Free Gibbs energies at 367.15 K.

Figure S1. Predicted conformers of cycloundecanone and their relative energies including zero-point corrections within 1000 cm^{-1}.

	**I**	**II**	**III**	**IV**	**V**
	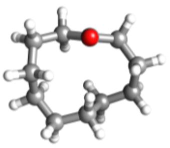	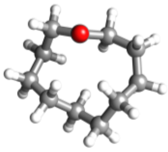	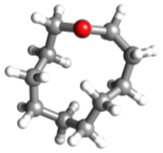	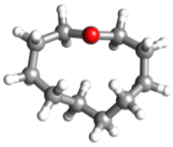	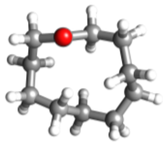
**ΔE_0 (B3LYP)**	0.0	250.4	355.3	532.9	562.3
**ΔE_0 (MP2)**	0.0	431.1	450.8	643.9	754.6

	**VI**	**VII**	**VIII**	**IX**	**X**
	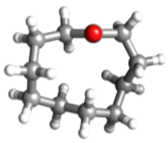	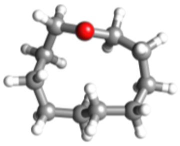	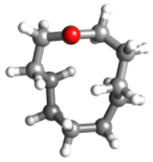	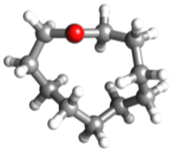	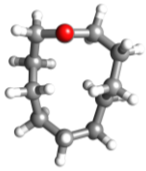
**ΔE_0 (B3LYP)**	580.3	629.0	652.1	710.4	714.6
**ΔE_0 (MP2)**	790.3	764.0	784.4	967.7	675.8

	**XI**	**XII**	**XIII**	**XIV**	**XV**
	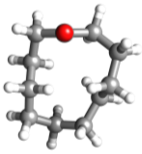	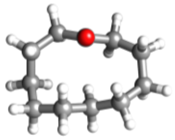	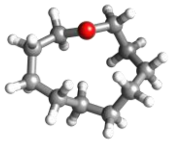	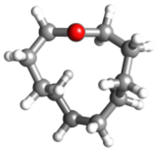	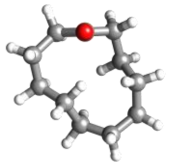
**ΔE_0 (B3LYP)**	736.8	749.3	767.5	856.8	859.7
**ΔE_0 (MP2)**	707.8	821.3	994.9	832.7	830.7

Table S3. Experimental spectroscopic constants of the ^{13}C isotopologues of conformer **I** of cycloundecanone.

Parameter	^{13}C1	^{13}C2	^{13}C3	^{13}C4	^{13}C5
A^{a} (MHz)	1070.61474(99)[d]	1074.6889(13)	1071.87157(82)	1068.0651(16)	1066.26487(84)
B (MHz)	868.31556(80)	863.7285(10)	864.46362(65)	871.4715(14	873.01809(67)
C (MHz)	568.0846(76)	567.1682(95)	566.6865(60)	568.948(12)	569.1240(67)
σ^{b} (kHz)	5.5	7.4	4.6	8.3	4.7
N^{c}	11	11	12	10	12

Parameter	^{13}C6	^{13}C7	^{13}C8	^{13}C9	^{13}C10	^{13}C11
A (MHz)	1063.5509(12)	1071.6359(16)	1074.6050(12)	1070.23351(86)	1067.05890(83)	1069.3991(12)
B (MHz)	870.85511(97)	866.6977(16)	865.35317(98)	866.55587(68)	871.48832(83)	873.39784(99)
C (MHz)	567.0483(97)	567.688(15)	567.9175(91)	567.1352(64)	569.3125(82)	569.6774(99)
σ (kHz)	6.6	8.1	7.2	4.8	3.9	7.2
N	9	10	11	12	7	12

[a] *A, B* and *C* are the rotational constants. Quartic centrifugal distortion constant fixed to the values of the parent species. [b] σ is the rms deviation of the fit. [c] *N* is the number of the fitted transitions. [d] Standard error in parentheses in units of the last digit.

Table S4. Substitution coordinates of the heavy atoms of conformer **I** of cycloundecanone in Å.

	a	*b*	*c*
C_1	1.81982(85)[a]	-1.3428(12)	0.4235(37)
C_2	2.5557(16)	-0.159(27)	-0.332(12)
C_3	2.44397(63)	1.1589(13)	0.3180(48)
C_4	1.0208(47)	1.6488(30)	0.6047(82)
C_5	0.124(24)	1.8624(16)	-0.6241(49)
C_6	-1.2957(31)	2.2510(19)	-0.291(14)
C_7	-2.0963(30)	1.1592(56)	0.433(15)
C_8	-2.3182(17)	-0.062(65)	-0.4098(96)
C_9	-2.1299(14)	-1.4395(21)	0.3302(90)
C_{10}	-0.7350(47)	-1.6263(22)	0.9347(38)
C_{11}	0.388(11)	-1.5727(26)	0.000(59)[b]

[a] Sign in accordance to B3LYP-D3BJ/6-311++G(d,p). The errors include Costain's error. [b] Imaginary coordinate set to zero.

Table S5. B3LYP-D3BJ/6-311++G(d,p) equilibrium structural parameters of all observed conformers of cycloundecanone. Bond lengths in Å, angles and dihedral angles in degrees (°).

**Conformer**	**I**	**II**	**III**	**IV**	**V**	**VI**	**VII**	**VIII**	**IX**
$r(C_1\text{-}C_2)$	1.545	1.534	1.544	1.534	1.542	1.545	1.540	1.536	1.545
$r(C_2\text{-}C_3)$	1.538	1.538	1.535	1.539	1.536	1.550	1.546	1.536	1.539
$r(C_3\text{-}C_4)$	1.534	1.549	1.537	1.552	1.535	1.537	1.538	1.535	1.537
$r(C_4\text{-}C_5)$	1.532	1.537	1.540	1.537	1.535	1.535	1.533	1.538	1.535
$r(C_5\text{-}C_6)$	1.538	1.535	1.540	1.536	1.540	1.534	1.532	1.541	1.540
$r(C_6\text{-}C_7)$	1.538	1.534	1.537	1.537	1.540	1.537	1.536	1.540	1.542
$r(C_7\text{-}C_8)$	1.545	1.534	1.536	1.541	1.550	1.538	1.537	1.537	1.541
$r(C_8\text{-}C_9)$	1.537	1.537	1.539	1.538	1.536	1.539	1.545	1.536	1.538
$r(C_9\text{-}C_{10})$	1.533	1.544	1.538	1.533	1.530	1.532	1.544	1.551	1.529
$r(C_{10}\text{-}C_{11})$	1.519	1.523	1.527	1.524	1.523	1.516	1.520	1.521	1.520
$r(C_{11}\text{-}C_1)$	1.526	1.523	1.519	1.522	1.522	1.523	1.523	1.527	1.523
$\angle(C_1\text{-}C_2\text{-}C_3)$	114.5	114.8	113.3	114.3	114.9	115.5	115.8	113.1	114.5
$\angle(C_2\text{-}C_3\text{-}C_4)$	116.0	116.0	114.1	116.5	115.4	114.3	116.6	114.6	113.1
$\angle(C_3\text{-}C_4\text{-}C_5)$	115.0	113.8	117.4	113.7	113.9	114.1	116.5	114.2	114.5
$\angle(C_4\text{-}C_5\text{-}C_6)$	113.8	113.6	116.8	113.7	115.5	113.4	114.3	117.6	115.9
$\angle(C_5\text{-}C_6\text{-}C_7)$	114.7	113.4	116.8	113.2	115.3	114.6	114.1	116.8	116.0
$\angle(C_6\text{-}C_7\text{-}C_8)$	113.8	114.3	114.7	114.5	117.1	117.3	114.5	116.6	120.3
$\angle(C_7\text{-}C_8\text{-}C_9)$	115.4	115.9	114.0	116.1	114.4	117.4	113.9	114.6	116.7
$\angle(C_8\text{-}C_9\text{-}C_{10})$	114.4	115.2	113.9	115.5	114.2	117.5	113.9	114.2	116.9
$\angle(C_9\text{-}C_{10}\text{-}C_{11})$	114.9	117.0	115.8	114.7	114.4	115.9	112.1	110.8	115.8
$\angle(C_{10}\text{-}C_{11}\text{-}C_1)$	118.2	116.8	116.7	116.9	117.3	117.4	116.5	119.2	116.3
$\angle(C_{11}\text{-}C_1\text{-}C_2)$	113.1	116.6	110.9	115.7	117.1	112.4	116.7	112.5	113.6
$\tau(C_1\text{-}C_2\text{-}C_3\text{-}C_4)$	-54.1	-76.2	-162.5	-79.8	-71.0	-121.2	-93.9	73.1	87.3
$\tau(C_2\text{-}C_3\text{-}C_4\text{-}C_5)$	-63.0	129.4	60.0	125.1	-69.3	68.5	61.2	-163.4	-163.6
$\tau(C_3\text{-}C_4\text{-}C_5\text{-}C_6)$	175.6	-69.6	66.0	-75.0	168.5	73.2	62.2	59.8	71.7
$\tau(C_4\text{-}C_5\text{-}C_6\text{-}C_7)$	-64.8	-72.3	-76.8	-79.3	-86.5	-170.4	-175.2	68.5	83.1
$\tau(C_5\text{-}C_6\text{-}C_7\text{-}C_8)$	-65.0	167.9	-66.5	171.3	75.4	55.9	62.3	-76.7	-64.2
$\tau(C_6\text{-}C_7\text{-}C_8\text{-}C_9)$	136.2	-63.5	163.8	-91.8	-125.9	69.3	62.6	-66.9	-74.7
$\tau(C_7\text{-}C_8\text{-}C_9\text{-}C_{10})$	-59.5	-63.7	-84.7	61.3	67.9	-70.0	-134.1	160.1	73.0
$\tau(C_8\text{-}C_9\text{-}C_{10}\text{-}C_{11})$	-58.9	75.6	74.4	-82.0	72.6	-59.2	61.8	-85.3	70.1
$\tau(C_9\text{-}C_{10}\text{-}C_{11}\text{-}C_1)$	163.0	65.5	-135.2	158.1	-167.2	168.6	75.9	88.1	-167.3
$\tau(C_{10}\text{-}C_{11}\text{-}C_1\text{-}C_2)$	-129.1	-164.4	73.8	-157.2	60.3	-105.8	-161.3	-138.1	105.0
$\tau(C_{11}\text{-}C_1\text{-}C_2\text{-}C_3)$	92.8	85.6	74.2	79.5	66.5	77.8	104.0	63.8	-58.2

Fig. S2. NCI isosurfaces (*s* = 0.5), for values of sign(λ_2)ρ ranging from −0.025 to +0.025 a.u, and plots of the reduced density gradient (RDG) versus sign(λ2)ρ, for the observed conformers of cycloundecanone. Blue indicates strong attractive interaction; green indicates weak attractive interaction; and red indicates strong repulsive interaction.

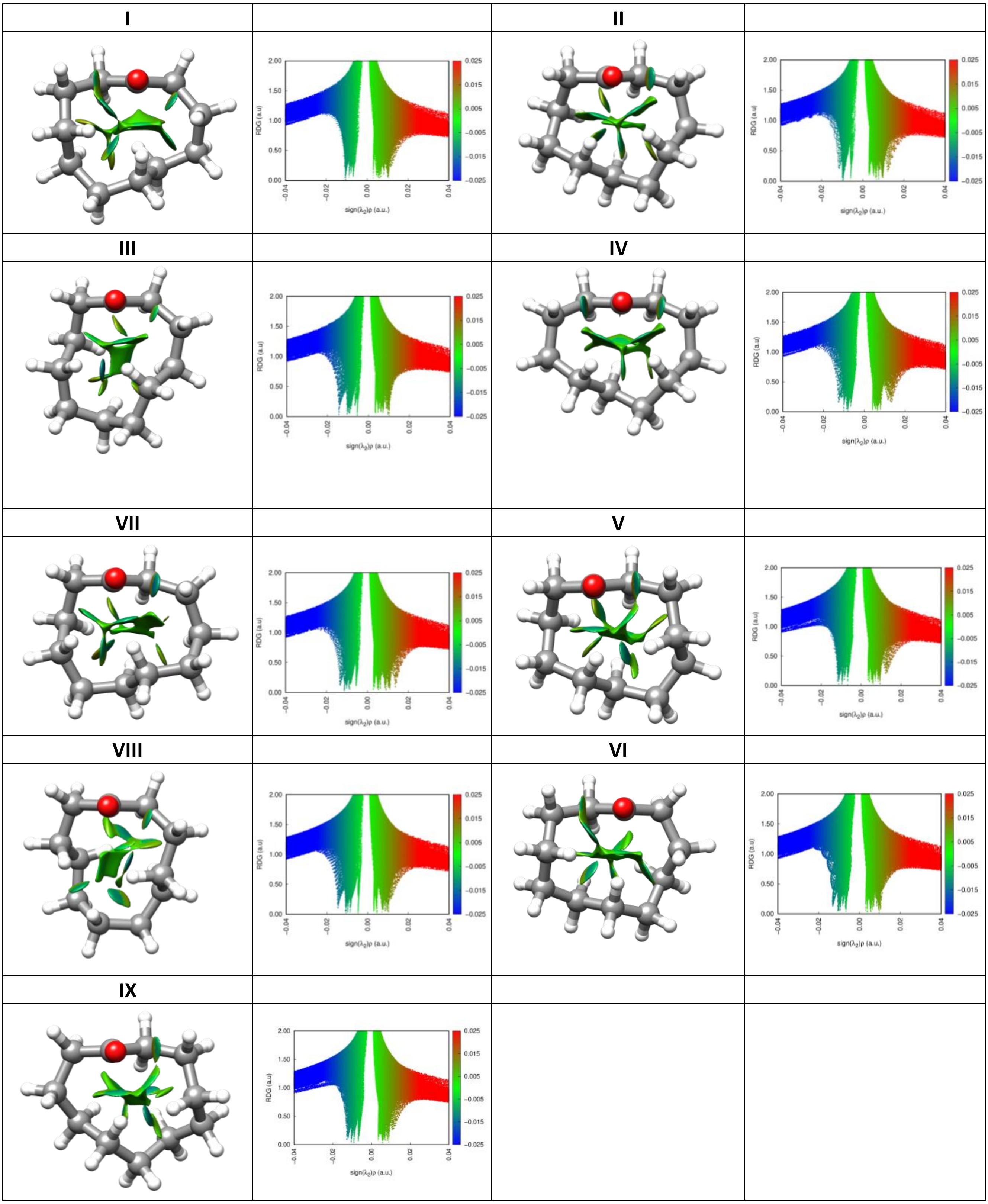

Fig. S3. Observed conformers of cycloundecanone, in decreasing order of abundance, showing their attractive C–H···O interactions. Distances are in Å.

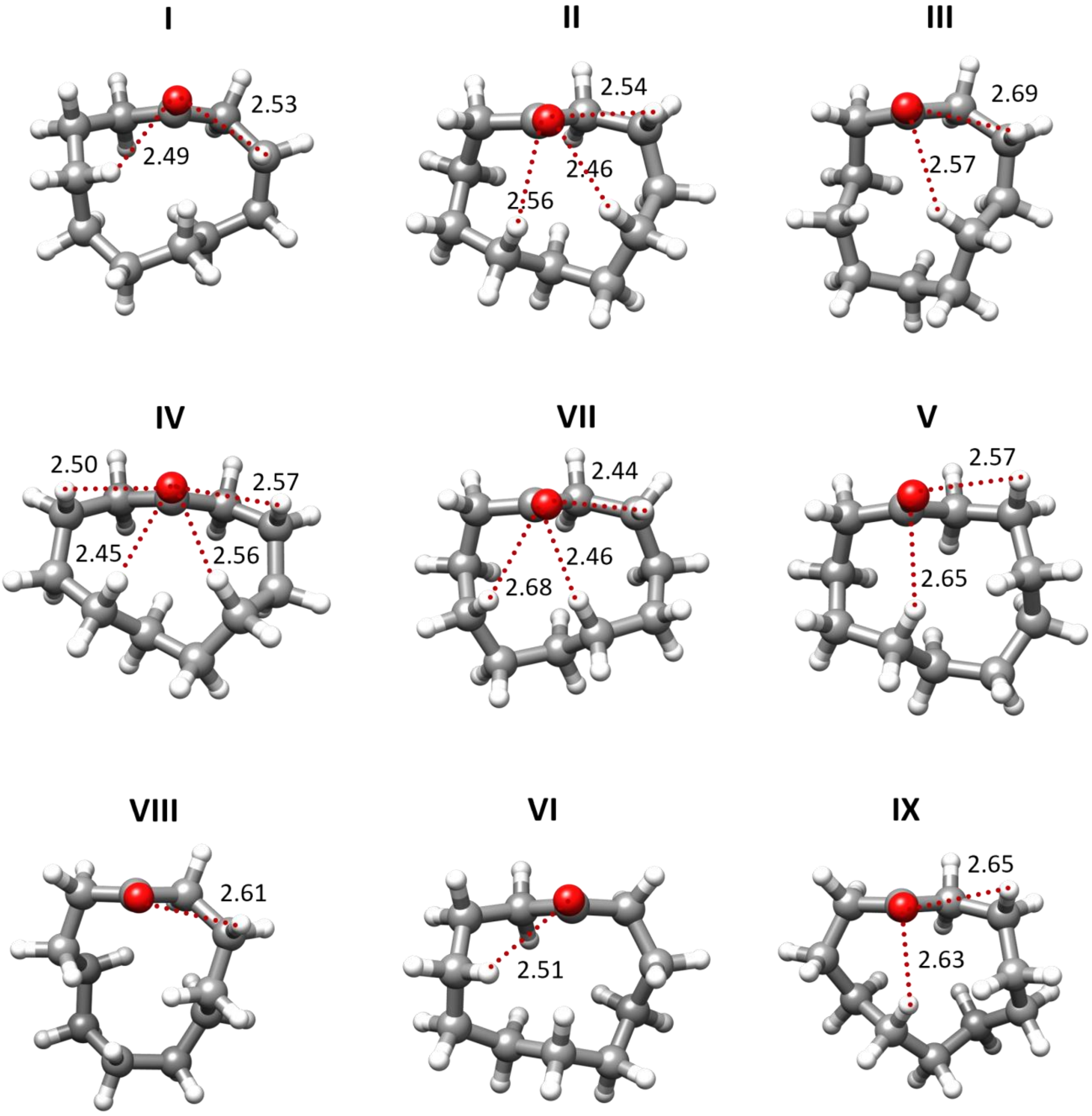

Table S6. Measured frequencies and residuals (in MHz) for the rotational transitions of conformer **I** of cycloundecanone.

J'	*K'₋₁*	*K'₊₁*	*J"*	*K"₋₁*	*K"₊₁*	ν_{obs}	ν_{obs}-ν_{calc}
2	1	2	1	1	1	2587.7189	0.0096
2	0	2	1	0	1	2716.8426	0.0014
2	1	2	1	0	1	2789.0879	0.0088
2	1	1	1	1	0	3192.2146	-0.0119
2	1	1	1	0	1	3695.8535	-0.0017
3	0	3	2	1	2	3774.2968	-0.0028
4	2	3	3	3	1	3781.5450	0.0165
3	1	3	2	1	2	3791.7957	0.0004
2	2	1	1	1	0	3796.3366	0.0009
3	1	2	2	2	0	3846.2465	0.0094
3	0	3	2	0	2	3846.5365	-0.0009
3	1	3	2	0	2	3864.0196	-0.0135
2	2	0	1	1	0	3969.4631	0.0006
2	2	1	1	1	1	4098.5972	0.0026
2	2	0	1	1	1	4271.7315	0.0101
3	2	2	2	2	1	4334.9438	-0.0056
3	1	2	2	1	1	4623.4727	-0.0004
3	2	1	2	2	0	4823.3559	-0.0062
4	1	3	3	2	1	4860.1131	-0.0043
3	2	2	2	1	1	4939.0685	0.0098
4	0	4	3	1	3	4951.6726	-0.0059
4	1	4	3	1	3	4955.1593	0.0016
4	0	4	3	0	3	4969.1706	-0.0037
4	1	4	3	0	3	4972.6571	0.0037
5	3	3	4	4	1	5099.9544	0.0051
6	2	5	5	3	3	5250.1836	-0.0080
5	1	4	4	2	2	5376.7069	0.0022
4	1	3	3	2	2	5521.6523	-0.0046
3	2	1	2	1	1	5600.5995	0.0014
3	1	2	2	0	2	5602.4861	-0.0009
6	1	5	5	2	3	5620.7739	0.0023
4	2	3	3	2	2	5633.7903	-0.0003
7	2	6	6	3	4	5714.3855	0.0007
4	1	3	3	1	2	5837.2422	-0.0002
3	2	2	2	1	2	5845.8376	0.0028
4	2	3	3	1	2	5949.3784	0.0022
3	3	1	2	2	0	6014.0860	0.0012
3	3	0	2	2	0	6089.1834	0.0021
5	0	5	4	1	4	6102.2947	-0.0098
5	1	5	4	1	4	6102.9304	0.0029
5	0	5	4	0	4	6105.7751	-0.0084
5	1	5	4	0	4	6106.4171	0.0105
8	1	7	7	2	5	6141.0009	-0.0016
3	3	1	2	2	1	6187.2131	0.0015
6	3	4	5	4	2	6207.6946	0.0017
3	3	0	2	2	1	6262.3052	-0.0029
4	3	1	3	3	0	6327.1241	-0.0057
4	2	2	3	2	1	6417.6414	-0.0029
5	2	3	4	3	1	6624.3698	-0.0042
5	1	4	4	2	3	6822.0922	-0.0057
5	2	4	4	2	3	6851.6697	0.0033
5	1	4	4	1	3	6934.2297	-0.0020
5	2	4	4	1	3	6963.8013	0.0011
5	2	3	4	3	2	7026.4208	0.0055

7	3	5	6	4	3	7035.8971	0.0023
4	3	2	3	2	1	7190.9087	0.0010
4	2	2	3	1	2	7394.7702	0.0009
5	3	3	4	3	2	7403.5531	-0.0012
6	2	4	5	3	2	7499.7260	-0.0108
4	3	1	3	2	1	7592.9473	-0.0018
4	1	3	3	0	3	7593.1954	0.0034
4	2	3	3	1	3	7687.8340	0.0038
5	2	3	4	2	2	7799.6745	-0.0043
4	3	2	3	2	2	7852.4456	-0.0016
7	2	5	6	3	3	7869.9415	0.0100

Table S7. Measured frequencies and residuals (in MHz) for the rotational transitions of the ^{13}C1 isotopologue of conformer **I** of cycloundecanone.

J’	K'_{-1}	K'_{+1}	J”	K''_{-1}	K''_{+1}	ν_{obs}	ν_{obs}-ν_{calc}
2	1	1	1	0	1	3675.5657	0.0059
2	2	0	1	1	0	3951.0021	0.0047
3	2	1	2	1	1	5570.9903	-0.0019
3	2	2	2	1	2	5816.7911	0.0052
3	3	0	2	2	0	6062.6197	-0.0052
3	3	1	2	2	1	6160.0160	0.0001
4	2	2	3	1	2	7353.2127	-0.0090
4	1	3	3	0	3	7551.5048	0.0082
4	3	1	3	2	1	7555.3268	0.0049
4	2	3	3	1	3	7647.9879	-0.0069
4	3	2	3	2	2	7814.4670	-0.0013

Table S8. Measured frequencies and residuals (in MHz) for the rotational transitions of the ^{13}C2 isotopologue of conformer **I** of cycloundecanone.

J’	K'_{-1}	K'_{+1}	J”	K''_{-1}	K''_{+1}	ν_{obs}	ν_{obs}-ν_{calc}
2	1	1	1	0	1	3665.8763	0.0036
2	2	0	1	1	0	3955.9543	-0.0071
3	1	2	2	0	2	5552.0900	-0.0197
3	2	1	2	1	1	5561.8676	0.0010
3	3	0	2	2	0	6078.3815	-0.0016
3	3	1	2	2	1	6174.5688	0.0025
4	2	2	3	1	2	7329.3620	0.0058
4	1	3	3	0	3	7530.3740	0.0034
4	3	1	3	2	1	7555.0205	-0.0014
4	2	3	3	1	3	7638.2983	0.0093
4	3	2	3	2	2	7817.3853	-0.0019

Table S9. Measured frequencies and residuals (in MHz) for the rotational transitions of the ^{13}C3 isotopologue of conformer **I** of cycloundecanone.

J’	K'_{-1}	K'_{+1}	J”	K''_{-1}	K''_{+1}	ν_{obs}	ν_{obs}-ν_{calc}
2	1	1	1	0	1	3665.2509	-0.0099
2	2	0	1	1	0	3949.3714	0.0046
3	1	2	2	0	2	5552.8743	0.0038
3	2	1	2	1	1	5558.7538	0.0026
3	2	2	2	1	2	5808.9969	-0.0036
3	3	0	2	2	0	6065.1302	0.0009
3	3	1	2	2	1	6161.7331	-0.0014
4	2	2	3	1	2	7329.8735	0.0007
4	1	3	3	0	3	7529.7893	0.0045
4	3	1	3	2	1	7546.0570	0.0018
4	2	3	3	1	3	7633.0559	-0.0084
4	3	2	3	2	2	7807.0428	0.0024

Table S10. Measured frequencies and residuals (in MHz) for the rotational transitions of the ^{13}C4 isotopologue of conformer **I** of cycloundecanone.

J’	K'_{-1}	K'_{+1}	J”	K''_{-1}	K''_{+1}	ν_{obs}	ν_{obs}-ν_{calc}
2	1	1	1	0	1	3682.4668	-0.0111
2	2	0	1	1	0	3948.4084	0.0158
3	2	1	2	1	1	5577.9786	0.0041
3	1	2	2	0	2	5583.9559	-0.0053
3	2	2	2	1	2	5818.6152	0.0105
3	3	0	2	2	0	6053.2475	0.0005
3	3	1	2	2	1	6151.2835	-0.0116
4	3	1	3	2	1	7557.1221	-0.0044
4	1	3	3	0	3	7565.9568	0.0035
4	3	2	3	2	2	7813.7380	-0.0002

Table S11. Measured frequencies and residuals (in MHz) for the rotational transitions of the ^{13}C5 isotopologue of conformer **I** of cycloundecanone.

J’	K'_{-1}	K'_{+1}	J”	K''_{-1}	K''_{+1}	ν_{obs}	ν_{obs}-ν_{calc}
2	1	1	1	0	1	3685.3239	0.0064
2	2	0	1	1	0	3945.6766	-0.0057
3	2	1	2	1	1	5580.2955	0.0044
3	1	2	2	0	2	5589.7200	-0.0004
3	2	2	2	1	2	5817.8460	0.0022
3	3	0	2	2	0	6045.9676	0.0016
3	3	1	2	2	1	6144.3762	0.0082
4	2	2	3	1	2	7377.5639	0.0032
4	3	1	3	2	1	7555.9018	-0.0018
4	1	3	3	0	3	7571.8396	-0.0014
4	2	3	3	1	3	7657.2061	-0.0052
4	3	2	3	2	2	7810.8998	-0.0071

Table S12. Measured frequencies and residuals (in MHz) for the rotational transitions of the ^{13}C6 isotopologue of conformer **I** of cycloundecanone.

J'	K'_{-1}	K'_{+1}	J"	$K"_{-1}$	$K"_{+1}$	ν_{obs}	ν_{obs}-ν_{calc}
2	2	0	1	1	0	3935.6122	-0.0045
2	2	1	1	1	1	4061.4927	-0.0133
3	2	1	2	1	1	5566.2110	-0.0072
3	3	0	2	2	0	6030.3864	0.0008
3	3	1	2	2	1	6128.7499	0.0031
4	2	2	3	1	2	7359.3659	0.0030
4	3	1	3	2	1	7536.5431	-0.0007
4	1	3	3	0	3	7553.2849	-0.0020
4	3	2	3	2	2	7791.1544	0.0110

Table S13. Measured frequencies and residuals (in MHz) for the rotational transitions of the ^{13}C7 isotopologue of conformer **I** of cycloundecanone.

J'	K'_{-1}	K'_{+1}	J"	$K"_{-1}$	$K"_{+1}$	ν_{obs}	ν_{obs}-ν_{calc}
2	1	1	1	0	1	3671.7363	0.0089
2	2	0	1	1	0	3951.6343	0.0086
2	2	1	1	1	1	4081.6113	0.0076
3	1	2	2	0	2	5563.8720	0.0004
3	2	1	2	1	1	5566.8756	-0.0045
3	3	0	2	2	0	6066.1152	-0.0035
3	3	1	2	2	1	6163.1270	-0.0001
4	1	3	3	0	3	7543.3155	-0.0085
4	2	3	3	1	3	7643.2870	0.0139
4	3	2	3	2	2	7813.5790	-0.0122

Table S14. Measured frequencies and residuals (in MHz) for the rotational transitions of the ^{13}C8 isotopologue of conformer **I** of cycloundecanone.

J'	K'_{-1}	K'_{+1}	J"	$K"_{-1}$	$K"_{+1}$	ν_{obs}	ν_{obs}-ν_{calc}
2	1	1	1	0	1	3670.6605	-0.0024
2	2	0	1	1	0	3957.8285	0.0093
2	2	1	1	1	1	4089.1468	-0.0198
3	1	2	2	0	2	5560.2329	0.0053
3	2	1	2	1	1	5567.9321	-0.0047
3	3	0	2	2	0	6079.5138	0.0035
3	3	1	2	2	1	6175.9995	0.0045
4	2	2	3	1	2	7339.8479	-0.0016
4	1	3	3	0	3	7540.4611	0.0011
4	3	1	3	2	1	7560.6758	-0.0005
4	3	2	3	2	2	7822.5332	0.0010

Table S15. Measured frequencies and residuals (in MHz) for the rotational transitions of the ^{13}C9 isotopologue of conformer **I** of cycloundecanone.

J'	K'_{-1}	K'_{+1}	J"	K''_{-1}	K''_{+1}	ν_{obs}	ν_{obs}-ν_{calc}
2	1	1	1	0	1	3669.8942	-0.0053
2	2	0	1	1	0	3947.6880	-0.0058
3	1	2	2	0	2	5561.7008	0.0045
3	2	1	2	1	1	5563.3849	0.0019
3	2	2	2	1	2	5810.3519	-0.0112
3	3	0	2	2	0	6059.0299	0.0012
3	3	1	2	2	1	6156.1710	0.0045
4	2	2	3	1	2	7341.1139	0.0030
4	1	3	3	0	3	7539.8120	-0.0041
4	3	1	3	2	1	7547.1190	0.0009
4	2	3	3	1	3	7638.1860	0.0039
4	3	2	3	2	2	7806.7170	0.0011

Table S16. Measured frequencies and residuals (in MHz) for the rotational transitions of the ^{13}C10 isotopologue of conformer **I** of cycloundecanone.

J'	K'_{-1}	K'_{+1}	J"	K''_{-1}	K''_{+1}	ν_{obs}	ν_{obs}-ν_{calc}
2	1	1	1	0	1	3681.5212	-0.0010
2	2	0	1	1	0	3945.8561	-0.0025
3	2	1	2	1	1	5576.0699	0.0058
3	3	0	2	2	0	6048.5781	-0.0016
3	3	1	2	2	1	6146.5060	0.0058
4	1	3	3	0	3	7563.6877	-0.0006
4	3	2	3	2	2	7809.3204	-0.0052

Table S17. Measured frequencies and residuals (in MHz) for the rotational transitions of the ^{13}C11 isotopologue of conformer **I** of cycloundecanone.

J'	K'_{-1}	K'_{+1}	J"	K''_{-1}	K''_{+1}	ν_{obs}	ν_{obs}-ν_{calc}
2	1	1	1	0	1	3689.5874	-0.0036
2	2	1	1	1	1	4081.6114	0.0178
3	2	1	2	1	1	5588.1450	-0.0020
3	1	2	2	0	2	5595.2769	0.0064
3	2	2	2	1	2	5828.3793	-0.0066
3	3	0	2	2	0	6061.5071	0.0071
3	3	1	2	2	1	6159.8996	-0.0103
4	2	2	3	1	2	7384.8990	0.0039
4	3	1	3	2	1	7569.5881	0.0019
4	1	3	3	0	3	7580.7605	-0.0009
4	2	3	3	1	3	7669.0678	-0.0047
4	3	2	3	2	2	7826.3245	-0.0034

Table S18. Measured frequencies and residuals (in MHz) for the rotational transitions of conformer **II** of cycloundecanone.

J'	K'_{-1}	K'_{+1}	J"	$K"_{-1}$	$K"_{+1}$	ν_{obs}	ν_{obs}-ν_{calc}
2	1	2	1	1	1	2621.7091	0.0072
2	0	2	1	0	1	2738.4015	-0.0040
2	1	1	1	1	0	3201.3393	0.0109
2	1	1	1	0	1	3666.8536	0.0003
3	1	3	2	1	2	3843.1701	0.0017
3	0	3	2	0	2	3888.9240	0.0008
2	2	0	1	1	0	3901.5676	-0.0039
2	2	1	1	1	1	4018.2733	-0.0021
3	2	2	2	2	1	4367.2708	0.0009
3	1	2	2	1	1	4633.0366	-0.0178
3	2	1	2	2	0	4845.6148	-0.0030
4	1	4	3	1	3	5026.5872	0.0039
4	0	4	3	0	3	5037.3919	0.0016
5	3	3	4	4	1	5361.0919	-0.0122
3	2	1	2	1	1	5545.8627	0.0019
3	1	2	2	0	2	5561.5045	0.0022
4	2	3	3	2	2	5675.8411	0.0017
3	2	2	2	1	2	5763.8445	0.0011
4	1	3	3	1	2	5851.1856	0.0072
3	3	0	2	2	0	5966.1765	-0.0056
4	3	2	3	3	1	6039.8426	0.0028
3	3	1	2	2	1	6059.7361	-0.0052
5	1	5	4	1	4	6196.4793	0.0076
5	0	5	4	0	4	6198.4989	-0.0119
4	3	1	3	3	0	6374.4907	0.0027
4	2	2	3	2	1	6431.1818	0.0026
5	2	3	4	3	1	6736.4197	0.0021
5	2	4	4	2	3	6906.6359	0.0035
5	1	4	4	1	3	6971.6813	-0.0018
4	2	2	3	1	2	7343.9885	0.0028
5	3	3	4	3	2	7446.4094	0.0055
4	3	1	3	2	1	7495.0468	-0.0055
4	1	3	3	0	3	7523.7645	0.0070
6	2	4	5	3	2	7579.4436	0.0009
4	2	3	3	1	3	7596.5138	-0.0006
4	3	2	3	2	2	7732.3073	-0.0039
5	2	3	4	2	2	7800.2892	-0.0014

Table S19. Measured frequencies and residuals (in MHz) for the rotational transitions of conformer **III** of cycloundecanone.

J'	K'_{-1}	K'_{+1}	J"	K''_{-1}	K''_{+1}	ν_{obs}	ν_{obs}-ν_{calc}
2	1	2	1	1	1	2510.5378	0.0020
2	0	2	1	0	1	2653.0991	-0.0005
2	1	1	1	0	1	3546.8106	0.0028
3	1	3	2	1	2	3707.8582	0.0003
3	0	3	2	0	2	3797.9166	0.0043
2	2	0	1	1	0	3970.3174	-0.0009
2	2	1	1	1	1	4112.8851	0.0026
3	2	2	2	2	1	4142.4168	-0.0017
3	1	2	2	1	1	4428.3293	0.0053
3	2	1	2	2	0	4486.9129	-0.0118
4	1	3	3	2	1	4690.0878	0.0030
4	1	4	3	1	3	4869.2699	0.0033
4	0	4	3	0	3	4904.8331	0.0026
3	1	2	2	0	2	5322.0353	0.0031
4	2	3	3	2	2	5434.8136	0.0025
3	2	1	2	1	1	5444.5553	0.0044
4	3	2	3	3	1	5675.0221	0.0014
4	1	3	3	1	2	5706.3100	-0.0018
3	2	2	2	1	2	5744.7741	0.0088
4	3	1	3	3	0	5842.2633	-0.0186
6	1	5	5	2	3	5922.7352	0.0020
5	1	5	4	1	4	6010.6331	0.0017
5	2	3	4	3	1	6013.0379	-0.0001
5	0	5	4	0	4	6021.5586	-0.0034
4	2	2	3	2	1	6045.0897	0.0015
3	3	0	2	2	0	6167.4970	-0.0003
3	3	1	2	2	1	6243.2450	-0.0001
7	3	5	6	4	3	6610.0258	0.0092
5	2	4	4	2	3	6666.8894	0.0012
6	3	3	5	4	1	6824.7143	0.0037
5	1	4	4	1	3	6841.4639	-0.0066
5	3	3	4	3	2	7059.2995	-0.0062
4	2	2	3	1	2	7061.3164	0.0011
6	1	6	5	1	5	7143.8335	0.0003
6	0	6	5	0	5	7146.7839	-0.0037
5	4	1	4	4	0	7199.6914	0.0031
4	1	3	3	0	3	7230.4350	0.0033
6	2	4	5	3	2	7304.3750	-0.0051
4	2	3	3	1	3	7471.7255	0.0070
5	2	3	4	2	2	7490.7985	-0.0058
4	3	1	3	2	1	7522.8559	0.0014
4	3	2	3	2	2	7775.8448	-0.0025
6	2	5	5	2	4	7849.0184	0.0003
6	1	5	5	1	4	7927.0648	-0.0058

Table S20. Measured frequencies and residuals (in MHz) for the rotational transitions of conformer **IV** of cycloundecanone.

J’	K'_{-1}	K'_{+1}	J”	K''_{-1}	K''_{+1}	ν_{obs}	$\nu_{obs}-\nu_{calc}$
2	1	1	1	0	1	3638.3479	0.0041
2	2	0	1	1	0	4123.7721	0.0033
5	2	4	4	3	2	4149.7379	-0.0150
2	2	1	1	1	1	4279.5058	0.0134
4	1	3	3	2	1	4663.7418	-0.0017
6	2	5	5	3	3	4932.0267	0.0121
4	1	4	3	0	3	4944.9007	-0.0047
6	3	4	5	4	2	5340.9847	0.0074
3	1	2	2	0	2	5449.7299	0.0056
5	1	4	4	2	2	5470.3107	0.0016
3	2	1	2	1	1	5605.8943	0.0043
6	1	5	5	2	3	5882.1798	0.0093
5	2	3	4	3	1	5929.9244	-0.0007
3	2	2	2	1	2	5938.3887	0.0116
5	0	5	4	1	4	6018.2742	0.0006
5	1	5	4	0	4	6042.6862	-0.0011
3	3	1	2	2	0	6395.3326	-0.0100
3	3	0	2	2	0	6427.0294	-0.0007
3	3	1	2	2	1	6505.6691	0.0003
5	2	4	4	1	3	7075.1131	-0.0159
4	2	2	3	1	2	7239.0439	-0.0023
6	2	4	5	3	2	7281.7209	-0.0098
4	1	3	3	0	3	7409.4674	0.0019
4	3	2	3	2	1	7589.1203	0.0008
4	2	3	3	1	3	7699.9043	0.0031
4	3	1	3	2	1	7785.0521	-0.0063

Table S21. Measured frequencies and residuals (in MHz) for the rotational transitions of conformer **V** of cycloundecanone.

J'	K'_{-1}	K'_{+1}	J"	$K"_{-1}$	$K"_{+1}$	ν_{obs}	ν_{obs}-ν_{calc}
2	1	1	1	0	1	3639.4254	0.0016
3	0	3	2	1	2	3712.9171	-0.0033
3	1	3	2	0	2	3784.4669	0.0031
2	2	0	1	1	0	3876.9344	0.0038
2	2	1	1	1	1	3997.0559	0.0109
3	2	2	2	1	1	4813.9647	0.0076
4	1	3	3	2	2	5462.5282	-0.0046
3	2	1	2	1	1	5504.2184	0.0025
3	1	2	2	0	2	5524.9140	0.0001
3	2	2	2	1	2	5727.3555	0.0055
4	2	3	3	1	2	5807.3605	-0.0062
3	3	1	2	2	0	5843.4332	-0.0172
3	3	0	2	2	0	5929.7856	-0.0006
5	1	5	4	0	4	5992.2692	0.0040
3	3	1	2	2	1	6027.8020	0.0018
3	3	0	2	2	1	6114.1345	-0.0016
5	1	4	4	2	3	6713.4153	-0.0088
5	2	4	4	1	3	6816.9907	0.0086
4	3	2	3	2	1	6993.0842	-0.0088
4	2	2	3	1	2	7292.1762	-0.0030
4	3	1	3	2	1	7438.3402	-0.0035
4	1	3	3	0	3	7476.9667	0.0043
4	2	3	3	1	3	7547.8189	0.0023
4	3	2	3	2	2	7683.3508	-0.0010
6	1	5	5	2	4	7871.3847	-0.0028
6	2	5	5	1	4	7895.2210	0.0005
5	3	3	4	2	2	7956.1906	0.0100

Table S22. Measured frequencies and residuals (in MHz) for the rotational transitions of conformer **VI** of cycloundecanone.

J'	K'_{-1}	K'_{+1}	J"	$K"_{-1}$	$K"_{+1}$	ν_{obs}	ν_{obs}-ν_{calc}
2	1	1	1	0	1	3671.9812	-0.0043
3	0	3	2	1	2	3740.4719	0.0027
3	0	3	2	0	2	3798.5272	-0.0082
3	1	3	2	0	2	3811.1055	0.0103
2	2	0	1	1	0	3908.8075	-0.0054
2	2	1	1	1	1	4029.5150	0.0059
4	1	4	3	1	3	4899.4149	-0.0042
4	0	4	3	0	3	4909.7477	0.0020
4	1	4	3	0	3	4911.9752	-0.0036
4	1	3	3	2	2	5508.4992	0.0000
3	2	1	2	1	1	5552.4577	0.0052
3	1	2	2	0	2	5575.3339	0.0020
4	1	3	3	1	2	5767.3426	-0.0079
3	2	2	2	1	2	5776.1171	0.0001
4	2	3	3	1	2	5849.3305	0.0114
3	3	1	2	2	0	5888.0228	0.0023
3	3	0	2	2	0	5976.8989	0.0003
3	3	1	2	2	1	6076.1752	-0.0030
3	3	0	2	2	1	6165.0523	-0.0040
4	2	2	3	2	1	6396.8868	-0.0030
5	1	4	4	2	3	6765.6566	-0.0025
5	2	4	4	1	3	6866.8391	0.0040
4	3	2	3	2	1	7044.9372	-0.0035

4	2	2	3	1	2	7358.6397	0.0031
4	3	1	3	2	1	7501.2833	-0.0024
4	1	3	3	0	3	7544.1523	0.0054
4	2	3	3	1	3	7613.5519	-0.0039
4	3	2	3	2	2	7747.8374	0.0011

Table S23. Measured frequencies and residuals (in MHz) for the rotational transitions of conformer **VII** of cycloundecanone.

J’	K'_{-1}	K'_{+1}	J”	K''_{-1}	K''_{+1}	ν_{obs}	ν_{obs}-ν_{calc}
2	1	1	1	0	1	3647.0858	0.0001
2	2	0	1	1	0	3882.8935	-0.0013
2	2	1	1	1	1	4000.7132	0.0063
3	2	1	2	1	1	5516.1267	-0.0001
3	1	2	2	0	2	5533.1966	-0.0018
3	2	2	2	1	2	5735.8443	0.0050
3	3	0	2	2	0	5938.4741	-0.0061
3	3	1	2	2	1	6033.4007	-0.0030
4	2	2	3	1	2	7305.2936	-0.0039
4	3	1	3	2	1	7455.2068	0.0023
4	1	3	3	0	3	7486.7261	0.0001
4	2	3	3	1	3	7559.0431	0.0025
4	3	2	3	2	2	7694.9485	0.0008

Table S24. Measured frequencies and residuals (in MHz) for the rotational transitions of conformer **VIII** of cycloundecanone.

J’	K'_{-1}	K'_{+1}	J”	K''_{-1}	K''_{+1}	ν_{obs}	ν_{obs}-ν_{calc}
2	1	1	1	0	1	3470.6278	-0.0037
3	1	3	2	1	2	3569.3877	0.0030
5	3	3	4	4	1	3576.6303	-0.0052
3	0	3	2	0	2	3683.3328	-0.0002
2	2	0	1	1	0	4022.2285	-0.0101
2	2	1	1	1	1	4173.1947	-0.0004
6	3	4	5	4	2	4827.1814	0.0020
3	1	2	2	0	2	5166.5206	-0.0032
3	2	1	2	1	1	5396.2376	0.0004
3	2	2	2	1	2	5732.8651	-0.0017
6	1	5	5	2	3	5818.4741	-0.0108
5	0	5	4	0	4	5820.6037	0.0160
3	3	0	2	2	0	6299.2121	0.0023
3	3	1	2	2	1	6363.7072	0.0014
5	2	4	4	2	3	6429.5877	0.0063
4	2	2	3	1	2	6902.3510	-0.0034
4	1	3	3	0	3	7011.2035	-0.0028
4	2	3	3	1	3	7388.5496	0.0051
4	3	1	3	2	1	7573.8624	-0.0007
4	3	2	3	2	2	7812.7917	-0.0005

Table S25. Measured frequencies and residuals (in MHz) for the rotational transitions of conformer **IX** of cycloundecanone.

J’	K'_{-1}	K'_{+1}	J”	K''_{-1}	K''_{+1}	ν_{obs}	ν_{obs}-ν_{calc}
2	1	1	1	0	1	3623.0946	0.0015
3	0	3	2	1	2	3693.3946	-0.0098
2	2	1	1	1	0	3827.1230	0.0112
3	1	3	2	0	2	3827.1230	0.0112
2	2	1	1	1	1	4109.5659	-0.0036
4	0	4	3	1	3	4868.0146	0.0039
4	1	4	3	0	3	4905.8760	-0.0017
3	1	2	2	0	2	5469.6697	-0.0005
3	2	1	2	1	1	5520.3536	0.0046
3	2	2	2	1	2	5799.5005	0.0037
3	3	1	2	2	0	6077.1447	0.0079
3	3	0	2	2	0	6129.3142	-0.0093
3	3	1	2	2	1	6219.5766	-0.0022
3	3	0	2	2	1	6271.7660	0.0004
4	2	2	3	1	2	7228.8071	0.0000
4	1	3	3	0	3	7430.7520	-0.0031
4	3	1	3	2	1	7548.0856	-0.0079
4	2	3	3	1	3	7587.4656	0.0084
4	3	2	3	2	2	7816.6185	-0.0018

Table S26. Cartesian coordinates of conformer **I** of cycloundecanone from B3LYP-D3BJ/6-311++G(d,p).

Centre number	Atomic Number	Coordinates (Å)		
		X	Y	Z
1	6	-1.827450	-1.401621	-0.372580
2	6	-2.566201	-0.257024	0.356707
3	6	-2.453533	1.113993	-0.330019
4	6	-1.030373	1.604595	-0.623496
5	6	-0.158422	1.836667	0.614554
6	6	1.293338	2.223804	0.287235
7	6	2.103168	1.130477	-0.430736
8	6	2.333211	-0.136651	0.423352
9	6	2.127009	-1.471669	-0.310712
10	6	0.720242	-1.666957	-0.887485
11	6	-0.403224	-1.613015	0.133464
12	8	-0.194698	-1.767257	1.319112
13	1	-1.840736	-1.251001	-1.455721
14	1	-2.194261	-0.214207	1.383420
15	1	-3.627374	-0.510897	0.428515
16	1	-2.971211	1.853581	0.292255
17	1	-3.005446	1.074042	-1.276885
18	1	-1.092406	2.539976	-1.192372
19	1	-0.544575	0.890833	-1.294546
20	1	1.806674	2.488465	1.218988
21	1	1.599806	0.873255	-1.367214
22	1	3.068207	1.549492	-0.732043
23	1	3.350959	-0.121782	0.825026
24	1	1.674768	-0.126812	1.293396
25	1	2.339497	-2.287741	0.384971
26	1	2.846466	-1.559934	-1.132673
27	1	0.643469	-2.650997	-1.371333
28	1	0.507471	-0.948058	-1.682924
29	1	1.290751	3.130751	-0.329482
30	1	-0.164085	0.946049	1.248110
31	1	-0.610342	2.632542	1.217847
32	1	-2.346630	-2.345103	-0.171929

Table S27. Cartesian coordinates of conformer **II** of cycloundecanone from B3LYP-D3BJ/6-311++G(d,p).

Centre number	Atomic Number	Coordinates (Å)		
		X	Y	Z
1	6	-0.481532	-1.786049	-0.717140
2	6	-1.965763	-1.572484	-0.392728
3	6	-2.455925	-0.122114	-0.536017
4	6	-2.050595	0.847148	0.602711
5	6	-1.410986	2.153614	0.106768
6	6	-0.014996	1.960920	-0.501152
7	6	1.066848	1.652629	0.541628
8	6	2.397090	1.171637	-0.052243
9	6	2.347621	-0.160261	-0.816982
10	6	1.970711	-1.392017	0.033537
11	6	0.505219	-1.543366	0.417646
12	8	0.156326	-1.563186	1.579910
13	1	-0.185498	-1.209625	-1.598656
14	1	-2.163209	-1.937313	0.619141
15	1	-2.548459	-2.200614	-1.073724
16	1	-3.547260	-0.139806	-0.613344
17	1	-2.104816	0.267730	-1.499252
18	1	-1.374060	0.343906	1.294975
19	1	-2.938354	1.093744	1.192699
20	1	0.274868	2.859639	-1.057644
21	1	1.253989	2.559661	1.127480
22	1	0.702851	0.912903	1.257012
23	1	2.786666	1.939109	-0.732150
24	1	3.131691	1.078963	0.755937
25	1	3.341268	-0.345840	-1.236337
26	1	1.677632	-0.079702	-1.677435
27	1	2.551200	-1.404336	0.959122
28	1	2.235060	-2.292021	-0.536067
29	1	-0.061773	1.158314	-1.243820
30	1	-1.343046	2.864704	0.938477
31	1	-2.072384	2.614353	-0.636484
32	1	-0.313123	-2.836227	-0.995375

Table S28. Cartesian coordinates of conformer **III** of cycloundecanone from B3LYP-D3BJ/6-311++G(d,p).

Centre number	Atomic Number	Coordinates (Å)		
		X	Y	Z
1	6	-1.293245	-2.000803	-0.290209
2	6	0.164641	-1.791780	-0.753196
3	6	1.162046	-1.694572	0.409128
4	6	2.515480	-1.076138	0.025203
5	6	2.507341	0.361275	-0.528286
6	6	2.072928	1.475580	0.441588
7	6	0.566355	1.607928	0.714889
8	6	-0.270161	2.049067	-0.495272
9	6	-1.781210	1.802801	-0.335041
10	6	-2.224029	0.380468	-0.717113
11	6	-1.867872	-0.717186	0.282496
12	8	-2.048925	-0.566190	1.472780
13	1	-1.349676	-2.780646	0.472120
14	1	0.210768	-0.889407	-1.368613
15	1	0.452693	-2.616265	-1.412736
16	1	1.338084	-2.702344	0.801688
17	1	0.717870	-1.138656	1.236280
18	1	2.991598	-1.720828	-0.724284
19	1	3.169441	-1.105239	0.904794
20	1	2.436159	2.432698	0.047341
21	1	0.176568	0.675585	1.115486
22	1	0.419385	2.342177	1.514899
23	1	-0.092967	3.115624	-0.671664
24	1	0.071683	1.540058	-1.403637
25	1	-2.329100	2.501188	-0.974458
26	1	-2.091781	2.007260	0.693947
27	1	-1.847691	0.124216	-1.711566
28	1	-3.319280	0.349938	-0.783708
29	1	2.592726	1.328242	1.396032
30	1	1.906574	0.407539	-1.442345
31	1	3.530707	0.582226	-0.850435
32	1	-1.906003	-2.303678	-1.146501

Table S29. Cartesian coordinates of conformer **IV** of cycloundecanone from B3LYP-D3BJ/6-311++G(d,p).

Centre number	Atomic Number	Coordinates (Å)		
		X	Y	Z
1	6	-1.008596	-1.810840	-0.630059
2	6	-2.332007	-1.306997	-0.041631
3	6	-2.608506	0.186556	-0.289219
4	6	-1.882418	1.190157	0.645410
5	6	-1.058524	2.248608	-0.105429
6	6	0.255442	1.708140	-0.687931
7	6	1.374737	1.582790	0.357337
8	6	2.635430	0.849457	-0.140364
9	6	2.676188	-0.667719	0.105468
10	6	1.564460	-1.476613	-0.571910
11	6	0.239139	-1.491258	0.180829
12	8	0.192143	-1.337274	1.382922
13	1	-0.882689	-1.454980	-1.657388
14	1	-2.351797	-1.521809	1.030423
15	1	-3.139914	-1.889399	-0.495377
16	1	-3.687266	0.347650	-0.204181
17	1	-2.363823	0.414229	-1.334160
18	1	-1.238910	0.651044	1.342697
19	1	-2.628107	1.699917	1.262483
20	1	0.607389	2.369093	-1.488314
21	1	1.649568	2.597908	0.663837
22	1	1.000584	1.086206	1.256343
23	1	2.770071	1.048192	-1.211870
24	1	3.513536	1.277109	0.354257
25	1	2.633777	-0.863342	1.180593
26	1	3.641610	-1.044493	-0.246443
27	1	1.862690	-2.532358	-0.639031
28	1	1.404251	-1.149147	-1.603289
29	1	0.066598	0.744722	-1.172435
30	1	-0.826287	3.080946	0.569275
31	1	-1.674524	2.670924	-0.907827
32	1	-1.025978	-2.907905	-0.697984

Table S30. Cartesian coordinates of conformer **V** of cycloundecanone from B3LYP-D3BJ/6-311++G(d,p).

Centre number	Atomic Number	Coordinates (Å)		
		X	Y	Z
1	6	2.419485	-0.611500	-0.289067
2	6	2.217460	0.836768	-0.777919
3	6	1.752921	1.824593	0.303092
4	6	0.305782	1.643562	0.781370
5	6	-0.747714	2.016460	-0.270508
6	6	-2.191599	1.594950	0.060516
7	6	-2.573358	0.165039	-0.364206
8	6	-2.021834	-0.997832	0.499133
9	6	-1.251259	-2.065495	-0.292047
10	6	0.076306	-1.577053	-0.875234
11	6	1.178296	-1.371648	0.156406
12	8	1.104006	-1.853073	1.268386
13	1	3.122953	-0.632677	0.546686
14	1	1.531754	0.853644	-1.629916
15	1	3.180013	1.180127	-1.169468
16	1	1.874374	2.844013	-0.082398
17	1	2.427479	1.739717	1.162601
18	1	0.145112	2.269415	1.666227
19	1	0.162062	0.620657	1.127659
20	1	-2.872702	2.283521	-0.450662
21	1	-3.665542	0.098557	-0.379908
22	1	-2.263333	0.029007	-1.407581
23	1	-1.384892	-0.619693	1.300299
24	1	-2.855557	-1.492282	1.006131
25	1	-1.882117	-2.429592	-1.110516
26	1	-1.048696	-2.918969	0.359227
27	1	-0.052377	-0.661972	-1.459386
28	1	0.473084	-2.314666	-1.586387
29	1	-2.380598	1.725840	1.133221
30	1	-0.481450	1.590038	-1.243960
31	1	-0.712556	3.103167	-0.409093
32	1	2.864024	-1.190328	-1.109079

Table S31. Cartesian coordinates of conformer **VI** of cycloundecanone from B3LYP-D3BJ/6-311++G(d,p).

Centre number	Atomic Number	Coordinates (Å)		
		X	Y	Z
1	6	-1.741029	-1.696547	-0.283104
2	6	-2.481200	-0.431109	0.205228
3	6	-2.219507	0.854473	-0.619691
4	6	-1.628478	2.018270	0.191736
5	6	-0.190161	1.780969	0.671059
6	6	0.850135	1.875302	-0.452080
7	6	2.263341	1.417664	-0.056157
8	6	2.410293	-0.004549	0.510665
9	6	2.185670	-1.178739	-0.458172
10	6	0.755114	-1.427624	-0.947983
11	6	-0.274763	-1.707728	0.129185
12	8	0.036792	-1.944907	1.278456
13	1	-1.841789	-1.791875	-1.368650
14	1	-2.219521	-0.271381	1.255463
15	1	-3.551368	-0.652327	0.193514
16	1	-3.160015	1.187440	-1.068099
17	1	-1.562979	0.629367	-1.466343
18	1	-2.275157	2.201104	1.057441
19	1	-1.661675	2.932352	-0.412937
20	1	0.905609	2.917698	-0.787857
21	1	2.923432	1.525552	-0.925839
22	1	2.647074	2.114060	0.699505
23	1	3.436632	-0.096876	0.882554
24	1	1.771578	-0.134897	1.385755
25	1	2.530373	-2.089476	0.040192
26	1	2.826032	-1.045593	-1.338182
27	1	0.739171	-2.309609	-1.604972
28	1	0.381524	-0.615991	-1.576829
29	1	0.509892	1.312443	-1.324246
30	1	-0.134070	0.805923	1.161473
31	1	0.062456	2.513837	1.445656
32	1	-2.198025	-2.574793	0.180641

Table S32. Cartesian coordinates of conformer **VII** of cycloundecanone from B3LYP-D3BJ/6-311++G(d,p).

Centre number	Atomic Number	Coordinates (Å)		
		X	Y	Z
1	6	-0.685793	-1.701308	-0.705620
2	6	-2.086431	-1.288187	-0.217757
3	6	-2.552122	0.127180	-0.628444
4	6	-2.290286	1.258782	0.379213
5	6	-0.824774	1.533940	0.734950
6	6	0.039072	1.980250	-0.448708
7	6	1.529058	2.142416	-0.111144
8	6	2.224641	0.847799	0.339851
9	6	2.247553	-0.259118	-0.738262
10	6	1.853279	-1.655790	-0.210794
11	6	0.432497	-1.683506	0.327667
12	8	0.207937	-1.713316	1.519956
13	1	-0.378905	-1.123675	-1.582642
14	1	-2.121484	-1.398960	0.869988
15	1	-2.804874	-2.006434	-0.620459
16	1	-3.634015	0.092936	-0.792666
17	1	-2.119863	0.381946	-1.602799
18	1	-2.831711	1.025148	1.303241
19	1	-2.743447	2.176852	-0.015416
20	1	-0.343927	2.933468	-0.831759
21	1	2.051375	2.545781	-0.987145
22	1	1.636003	2.892375	0.681117
23	1	3.250216	1.083622	0.638759
24	1	1.736523	0.473191	1.242593
25	1	3.245449	-0.333375	-1.178729
26	1	1.582008	0.006675	-1.563928
27	1	2.521891	-1.957776	0.598215
28	1	1.940947	-2.384394	-1.024609
29	1	-0.070241	1.271012	-1.275080
30	1	-0.404091	0.643591	1.202967
31	1	-0.792276	2.314010	1.505040
32	1	-0.703225	-2.744212	-1.052040

Table S33. Cartesian coordinates of conformer **VIII** of cycloundecanone from B3LYP-D3BJ/6-311++G(d,p).

Centre number	Atomic Number	Coordinates (Å)		
		X	Y	Z
1	6	-1.818826	0.780003	-0.905543
2	6	-1.284992	2.061274	-0.247681
3	6	0.066910	1.860652	0.453204
4	6	1.261500	1.703494	-0.497707
5	6	2.519802	1.113050	0.161482
6	6	2.405746	-0.289876	0.788293
7	6	2.207431	-1.468940	-0.182386
8	6	0.798447	-1.645927	-0.770282
9	6	-0.275343	-2.041699	0.254035
10	6	-1.728180	-1.752759	-0.205110
11	6	-2.119586	-0.317808	0.112360
12	8	-2.641786	-0.045967	1.174041
13	1	-1.145576	0.407672	-1.679532
14	1	-2.022953	2.397513	0.483716
15	1	-1.201584	2.846672	-1.006754
16	1	-0.013685	0.990419	1.109885
17	1	0.257743	2.709808	1.118066
18	1	1.516187	2.688423	-0.905891
19	1	0.982772	1.099799	-1.364306
20	1	1.620928	-0.296900	1.550759
21	1	2.922298	-1.365620	-1.007448
22	1	2.481555	-2.394602	0.338295
23	1	0.505381	-0.730915	-1.280087
24	1	0.833857	-2.416345	-1.548845
25	1	-0.110957	-1.532637	1.207743
26	1	-0.184630	-3.110522	0.469193
27	1	-2.419498	-2.397580	0.340126
28	1	-1.827571	-1.957397	-1.276374
29	1	3.336900	-0.469377	1.336287
30	1	2.847983	1.802675	0.948872
31	1	3.328141	1.096682	-0.579607
32	1	-2.770457	0.991182	-1.410419

Table S34. Cartesian coordinates of conformer **IX** of cycloundecanone from B3LYP-D3BJ/6-311++G(d,p).

Centre number	Atomic Number	Coordinates (Å)		
		X	Y	Z
1	6	2.493172	-0.397736	-0.512199
2	6	2.491723	1.065155	-0.014460
3	6	1.201919	1.843779	-0.326020
4	6	0.120655	1.675186	0.752750
5	6	-1.289232	2.091055	0.311541
6	6	-1.983386	1.154797	-0.695166
7	6	-2.692975	-0.074767	-0.094526
8	6	-1.879993	-1.162094	0.633385
9	6	-1.054300	-2.114121	-0.248430
10	6	0.207328	-1.540539	-0.894218
11	6	1.355124	-1.236641	0.054059
12	8	1.386771	-1.657007	1.192107
13	1	3.428930	-0.874579	-0.208342
14	1	3.349069	1.562965	-0.476654
15	1	2.671890	1.070337	1.065287
16	1	0.820642	1.533149	-1.305301
17	1	1.432044	2.909668	-0.425817
18	1	0.414976	2.273628	1.622211
19	1	0.109590	0.646017	1.107148
20	1	-1.279921	0.856655	-1.477567
21	1	-3.442248	0.303400	0.611984
22	1	-3.263274	-0.561066	-0.896428
23	1	-1.247561	-0.728083	1.409542
24	1	-2.603387	-1.785014	1.170870
25	1	-1.701281	-2.503346	-1.043138
26	1	-0.755832	-2.971606	0.360835
27	1	0.003159	-0.648007	-1.489415
28	1	0.617687	-2.263563	-1.614666
29	1	-2.755038	1.733223	-1.215153
30	1	-1.230974	3.096133	-0.123535
31	1	-1.929180	2.184672	1.196835
32	1	2.446342	-0.402960	-1.605843
